# Cloud BioLinux: pre-configured and on-demand bioinformatics computing for the genomics community

**DOI:** 10.1186/1471-2105-13-42

**Published:** 2012-03-19

**Authors:** Konstantinos Krampis, Tim Booth, Brad Chapman, Bela Tiwari, Mesude Bicak, Dawn Field, Karen E Nelson

**Affiliations:** 1 J.Craig Venter Institute, 9704 Medical Center Drive, Rockville, MD 20850, USA; 2 CEH Wallingford, Benson Lane, Wallingford, UK; 3 Bioinformatics Core, Harvard School of Public Health, 655 Huntington Avenue, Boston, MA 02115, USA; 4 CLC Bio, Finlandsgade 10, 8200 Århus N, Denmark

## Abstract

**Background:**

A steep drop in the cost of next-generation sequencing during recent years has made the technology affordable to the majority of researchers, but downstream bioinformatic analysis still poses a resource bottleneck for smaller laboratories and institutes that do not have access to substantial computational resources. Sequencing instruments are typically bundled with only the minimal processing and storage capacity required for data capture during sequencing runs. Given the scale of sequence datasets, scientific value cannot be obtained from acquiring a sequencer unless it is accompanied by an equal investment in informatics infrastructure.

**Results:**

Cloud BioLinux is a publicly accessible Virtual Machine (VM) that enables scientists to quickly provision on-demand infrastructures for high-performance bioinformatics computing using cloud platforms. Users have instant access to a range of pre-configured command line and graphical software applications, including a full-featured desktop interface, documentation and over 135 bioinformatics packages for applications including sequence alignment, clustering, assembly, display, editing, and phylogeny. Each tool's functionality is fully described in the documentation directly accessible from the graphical interface of the VM. Besides the Amazon EC2 cloud, we have started instances of Cloud BioLinux on a private Eucalyptus cloud installed at the J. Craig Venter Institute, and demonstrated access to the bioinformatic tools interface through a remote connection to EC2 instances from a local desktop computer. Documentation for using Cloud BioLinux on EC2 is available from our project website, while a Eucalyptus cloud image and VirtualBox Appliance is also publicly available for download and use by researchers with access to private clouds.

**Conclusions:**

Cloud BioLinux provides a platform for developing bioinformatics infrastructures on the cloud. An automated and configurable process builds Virtual Machines, allowing the development of highly customized versions from a shared code base. This shared community toolkit enables application specific analysis platforms on the cloud by minimizing the effort required to prepare and maintain them.

## Background

High-throughput genomic technologies continue to move in a direction where data yield from the instruments is increasing, while the cost for acquiring the technology is continuously decreasing. For example, the introduction of benchtop genome sequencers such as MiSeq from Illumina [1], has made complete sequencing of viral, bacterial, and small fungal genomes affordable to small laboratories. Nonetheless, acquiring the sequence is only the first step, and must be followed by large-scale computational analysis to process the data, test hypotheses and draw scientific insights. Therefore, investment in a sequencing instrument would normally be accompanied by substantial investment in computer hardware, skilled informatics support, and bioinformaticians competent in configuring and using specific software to analyze the data.

An alternative model is now available: computational capacity can be purchased as a service from a cloud computing provider, and specialized computational systems can be run on such platforms [2]. Cloud infrastructures provide researchers with the ability to perform computations using a practically unlimited pool of Virtual Machines (VMs), without the burden of owning or maintaining hardware [3]. Cloud computing services use a charge model similar to utilities such as electricity, and thus customers are billed based on amounts of computing resources consumed [4]. Along these lines, the Cloud BioLinux project offers an on-demand, cloud computing solution for the bioinformatics community, and is available for use on private or publicly accessible, commercially hosted cloud computing infrastructure such as Amazon EC2. For small laboratories without access to large computational resources, running Cloud BioLinux through a commercial cloud platform provides a cost-effective route from data to knowledge, while those with access to private clouds will still benefit from the abundance of pre-configured software and the user-friendly desktop interface available.

Cloud BioLinux takes advantage of the fact that VMs provide a mechanism for whole system snapshot exchange [5]. With this approach, the operating system, software tools and databases, are encapsulated into a single digital image of the computing system that is readily archived and restored for later use. A snapshot captures all changes made inside a VM server from its initial execution, up to the point of snapshot creation. These changes include for example user-uploaded data, configuration settings and analysis results generated by running bioinformatic pipelines. A snapshot is also an executable VM, and can be shared with other users of the cloud, therefore allowing collaborating researchers to share uploaded data, analysis results and bioinformatics tools in as a single digital image. Having access to specialized VMs with scientific results for a particular scientific domain can greatly speed up research, as it substantially decreases, and in many cases removes the time required for an individual to configure the computing system with data and software to meet their research needs.

An early pioneering effort to provide such a system for the bioinformatics community was NEBC BioLinux [6]. NEBC BioLinux contains over 135 bioinformatics packages, including the blastall and blast+ NCBI applications, the Staden toolkit, EMBOSS, hmmer, and phylip collections of software, many stand-alone applications for tasks such as sequence alignment, clustering, assembly, display, editing, and phylogeny, as well as tools for working with next generation sequencing data. The system is also designed to allow setting up and maintaining a data analysis environment with very little informatics expertise, running a "live system" from a DVD or USB stick (without modifications to the user's workstation), or installing it to the hard drive with a simple graphical installer.

Building on the bioinformatics packages, documentation and desktop interface of NEBC BioLinux release 6.0, we developed Cloud BioLinux by leveraging VM technology and the cloud to offer a pre-configured, high-performance bioinformatics computing solution. Included by default are all bioinformatic software packages from NEBC BioLinux, in addition to next-generation sequencing data analysis tools including for example the Fastx utilities, SAM and BAM toolsets, Genome Analysis Toolkit (GATK), BWA, Novoalign and Bowtie aligners, the Mummer toolkit, and the Velvet, SSAKE, Mira, Newbler and Cap3 genome seqeunce assemblers. Furthermore, bioinformatic code libraries such as BioPython, BioPerl, BioRuby, BioJava, R and R-Bioconductor programming languages are included. Besides the pre-installed software, Cloud BioLinux provides scripts for accessing a repository of reference genomes (human, mouse, *D. melanogaster*, *A. thaliana*, *X. tropicallis, S. cerevisiae *and *C. elegans*) on an Amazon S3 bucket. The reference genomes are pre-indexed for a number of popular sequence alignment software packages, including BWA, Bowtie and Novoalign. A script and configuration files are included as part of the Cloud BioLinux framework, for selecting indexed genomes and installing them directly from the cloud storage on a running VM.

Detailed documentation for each tool included in Cloud BioLinux is available as set of HTML pages structured as mini-website, and linked from the main Cloud BioLinux website and as an icon on the graphical interface of the VM (in addition, readers can download the complete set of documentation as a compressed file from Additional file 1; Suppl.1). This mini, self-contained website allows users to select documentation for the installed packages from a drop-down menu, where the applications grouped based on their functionality, with some example groups including Statistics, Alignment, Clustering, Databases, Microarrays and Phylogeny.

End-users can simply instantiate Cloud BioLinux VMs using only a web browser through a local desktop computer to access the Amazon EC2 cloud console, and then login to the rich graphical interface using a remote connection without need for any advanced technical knowledge. An example remote desktop client is the one by NoMachine [7], which is available at no charge for Windows, Mac or Linux computers. For advanced users and developers, we have implemented an automated software management framework, which allows complete customization of the bioinformatics tools included in the Cloud BioLinux VM, while also enabling easy updates and deployment on different cloud platforms. Since the project is fully open-source, researchers and software developers at their laboratories can freely download, modify, and run the VMs on a public or private cloud.

In the following sections we first present the technical details of the Cloud BioLinux software management framework, and how it can be leveraged for creating customized VMs and deploying to different cloud platforms. Then we detail how end-users without access to local computing infrastructure, can run Cloud BioLinux by simply using a desktop computer with Internet access. Finally, we discuss our future plans for further development of this project.

## Implementation

The Cloud BioLinux project is community-centered and was designed for collaborative development, while the wide range of software included in the VM can serve a variety of bioinformatics analysis needs with a research groups from initial processing of next-generation seqeuncing data (Fastx utilities, SAM and BAM toolsets) to genome assembly (Velvet, SSAKE, Newbler), and sequence database search (HMMR, Blast). Nonetheless, in order to also support advanced users and developers towards building and distributing customized VM configurations on public or private cloud platforms, we implemented a modular system that bootstraps the Cloud BioLinux VM creation process and is freely available from our project's code repository on GitHub [8]. The code repository provides a focus for the collaborative developer community to contribute and review updates to the system, as well as being the basis for creating and distributing customised rebuilds of the VM.

**Figure 1:**
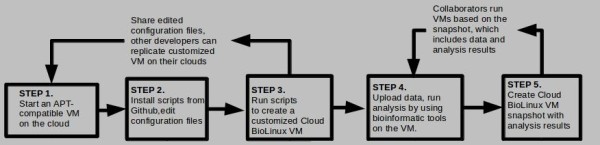
**Cloud computing workflows for sharing VMs and research data**. Steps 1,2,3: Developers create and share customized Cloud BioLinux VMs; Steps 4,5: Researchers exchange snapshots with data, run tools and collaboratively expand analysis results.

The system is composed of a main driver script and a set of configuration files that developers and advanced users can edit in order to select bioinformatics software and create their own customized version of Cloud BioLinux. The design goal for the system is to simplify the process of selecting the software configuration, automate building of new bioinformatic VMs with the specified software, and seamlessly deploy them on Amazon EC2, Eucalyptus or OpenStack clouds [9-11]. The implementation is based on the Fabric software management system [12] and Debian Aptitude [13]. For deploying a customized Cloud BioLinux, developers can start from a base VM containing any Debian Aptitude (APT) compatible Linux operating system, where the bioinformatics tools will be installed (Step 1, Figure 1). For deployment on Amazon EC2 we used the Ubuntu Alestic VMs [14]. A set of base VMs are freely available via the Ubuntu Enterprize Cloud website for download and use on a Eucalyptus clouds [15], but developers requiring non-default settings can create their own from scratch using Ubuntu builder [16].

Next (Step 2, Figure 1), software developers download the script and configuration files from our GitHub code repository, and then can edit the configuration files to customize the bioinformatics tools to be included in Cloud BioLinux. By default the software specified in the configuration files is from the APT-based NERC BioLinux 6.0 repository. In these configuration files, we have categorized software packages in modules based on their bioinformatics functionality, including for example next generation sequencing data analysis, *de novo *assembly, annotation, phylogeny, molecular modeling and gene expression analysis. During customization developers can add or remove complete modules, or individual software packages from each module. In addition to the BioLinux 6.0 software, extra packages are installed from the standard Ubuntu APT repository where they are maintained through the efforts of the Debian-Med community [17]. Additional APT compatible bioinformatic or scientific software repositories can be specified (a detailed list is available from [18]), and software can be mixed and matched from the different repositories including for example code libraries from the Comprehensive Perl Archive Network [19]. When the scripts are executed (Step 3, Figure 1) the configuration files are parsed by Fabric, which performs retrieval and installation of the selected software from the NERC or the other repositories, converting the base VM to a fully-functional, customized Cloud BioLinux system.

Within the same framework an additional Fabric script and configuration file, allow managing and accessing an Amazon S3 repository of pre-indexed reference genomes for a number of popular sequence alignment packages. Similarly with the scripts that install customized sets of software within Cloud BioLinux, a user can select reference genomes to install in the VM by editing the configuration file, and the Fabric script automatically downloads and installs the data during build time. Instructions for executing the script, are provided from the project's source code repository on GitHub [8].

Collaborating software developers can use a source code repository like GitHub where our original files reside, and share the edited configuration files in order to distribute their customized version of Cloud BioLinux with selected software and data. The process follows a circular workflow as shown on Steps 1,2,3 of Figure 1 where a developer starts from a base VM, retrieves and edits the configuration files, then runs the scripts to create a customized version of Cloud BioLinux; finally, she shares the edited configuration files by committing the changes back to the GitHub repository. Other developers can replicate the customized VM on their cloud by performing on more cycle: start a base VM, retrieve the edited configuration files and run the Fabric scripts. If desired, the developer can make additional customizations in the configuration files, which are also shared through the same cycle. This process can be repeated multiple times among more than two collaborators, and across any public or private cloud platform.

On the other hand, end-users using a customized or full-version of Cloud BioLinux prepared by developers, can leverage the flexibility of working with VMs on the cloud by sharing their research based on the circular workflow shown on the Steps 4,5 of Figure 1. In the first step a researcher can upload data from her local desktop computer such as genomic reads generated at her laboratory, or retrieve data from public databases such as NCBI (for example reference genomes) using the web browser or the standard FTP utilities available in the Cloud BioLinux VM. Then, data analysis such as a *de novo *genome assembly with Velvet or mapping reads to a reference genome using BWA, can be performed. Following analysis the researcher has the option to create a whole system snapshot and make the assemblies accessible to collaborators by granting them access to the snapshot on Amazon EC2, or exporting the snapshot for download and execution on private clouds. Using the snapshot, collaborators can execute new Cloud BioLinux VMs on EC2 or their private cloud that now include the genome reads and sequence assembly, then repeat the cycle by uploading additional data or re-creating the genome assembly with different parameters and generating a new snapshot to share their updates in turn.

## Results

### Cloud BioLinux Virtual Machines on the cloud

We have made Cloud BioLinux publicly available through the Amazon EC2 cloud, which is an industry-scale cloud platform backing the worldwide Amazon.com informatics infrastructure with data centers in US East and West regions, European Union and Asia. With this approach, we can enable researchers without access to local computing clusters to perform large-scale data analysis, by tapping into a pool of on-demand Cloud BioLinux VMs that can be rented at low cost starting from 0.085$ US per hour for a single core/1.7 GB memory RAM/160 GB of storage VM, and up to 2$ US for VMs with 8 cores/64 GB of RAM/1.68 TB of storage based on Amazon EC2 pricing [20], and are available worldwide and independently of institutional, economic or national boundaries.

**Figure 2:**
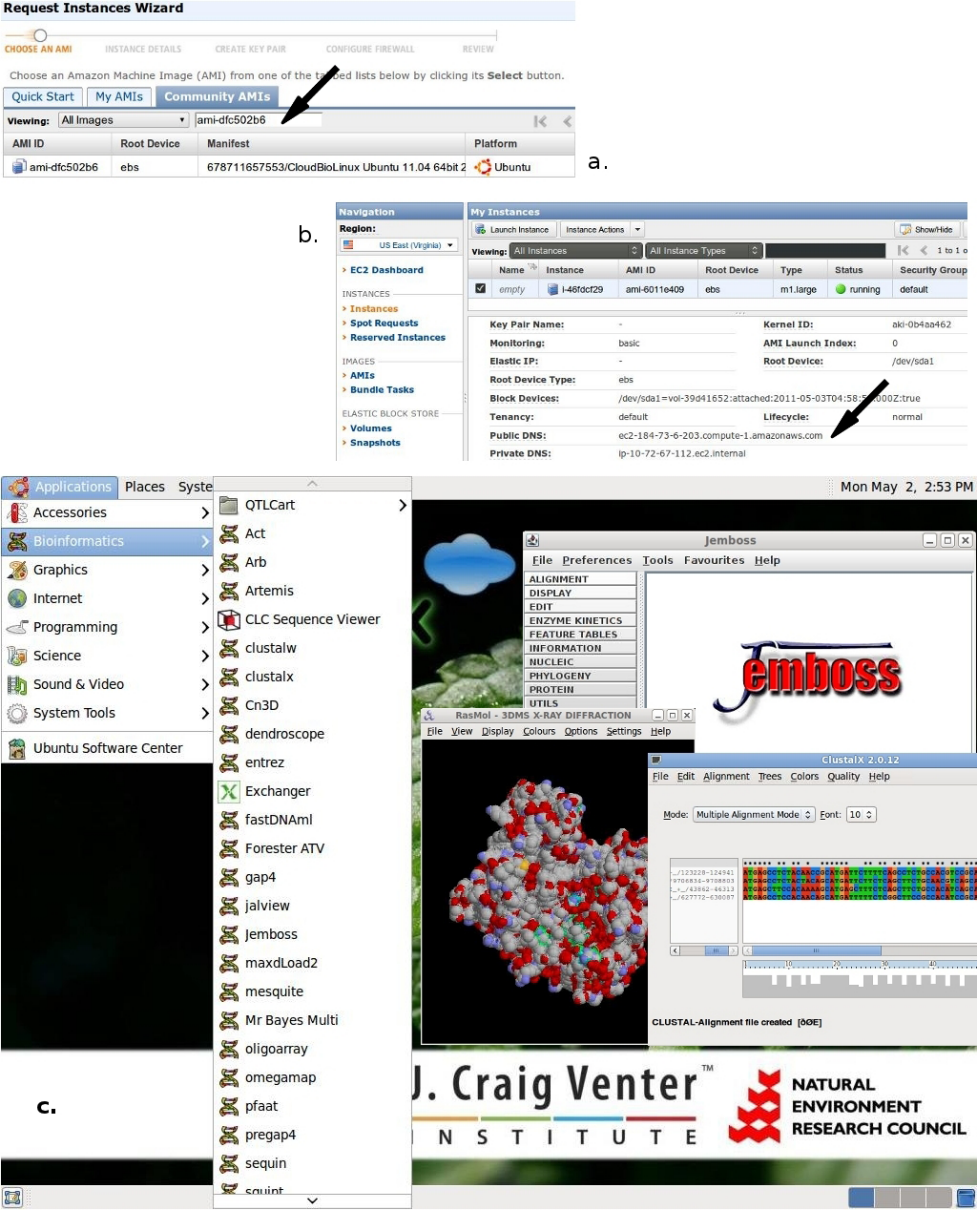
**Using Cloud BioLinux**. (a, b) Cloud BioLinux on Amazon EC2 cloud console, the arrows designate the Cloud BioLinux VM image identifier and internet address assigned to the VM respectively; (c) Cloud BioLinux remote desktop interface.

A user can run Cloud BioLinux in three simple steps through a web browser, assuming only access to a desktop computer with internet connection: 1. sign up for an Amazon EC2 account and login to the cloud console [21]; 2. within the console use the wizard (via "Launch Instance" button) for launching Cloud BioLinux and specify the Cloud BioLinux VM image identifier (Figure 2a, at the time of writing this is ami-cf945fa6, our project website provides the most recent update), then select computational capacity, storage, number of VMs to execute and specify a password for the remote desktop login; 3. finally, copy from the web browser window of the Amazon cloud console the assigned internet address to the VM ("Public DNS" on Figure 2b) for the launched Cloud BioLinux, and paste it in the remote desktop client. After the remote desktop client establishes the connection, a user has access to a full desktop session with applications presented in a bioinformatics menu, in addition to documents and tutorials linked via desktop icons (Figure 2c).

The steps for running Cloud BioLinux are described in more detail in the documentation available from our project's website. Also the procedure for transferring data from the local computer to the cloud is explained, including a troubleshooting guide for common problems. For higher security, instead of providing a password through the EC2 launch instance wizard a user can take the additional step of setting up Secure Shell (SSH) keys, which is fully described in the documentation. Alternatively the Cloud BioLinux VM can be executed on an open-source Eucalyptus cloud, or directly on a desktop computer using virtualization software like Virtualbox [22]. A Virtualbox Appliance to run on a common desktop computer (3 GB of RAM and dual-core processor is sufficient for Virtualbox Appliances) is available on our project website, in addition to a VM ready to execute on the Eucalyptus cloud.

### Next-gen sequencing data analysis with Cloud BioLinux

The goal of the Cloud BioLinux project is to provide a configurable, automated framework for building VMs with biological software. Currently, producing a push button analysis platform for a particular analysis community requires both expertise and effort to move from a base VM to a fully configured cloud computing solution. Projects like CloVR, Bioconductor, Qiime and GMOD [23-26] demonstrate the usefulness of pre-configured VMs with specific types of analysis for biologists; by establishing CloudBioLinux as a community framework we hope to ease the production of these resources for a broad audience of bioinformatics developers from various backgrounds and with different research goals.

Overall, bioinformatics pipelines that involve datasets that can be segmented and their parts independently processed (horizontal scaling), are good candidates for parallelization on a cloud computing environment. The reason is that currently available cloud computing architectures such as Amazon EC2 do not offer extremely large-memory VMs (the maximum allowable memory on EC2 is 64 GigaBytes) for vertically-scaling, monolithic software applications such as de novo genome assembly. This makes the cloud approach only feasible for the assembly of smaller genomes. Unlike *de novo *assembly, horizontally scaling software such as mapping assemblies, sequence database search, gene finding and genome annotation are well suited for execution on the cloud.

Based on these principles, we have prepared a use-case example by developing a parallel exome sequencing data analysis pipeline (Additional file 2; Suppl. 2). The pipeline is composed of a set of Python scripts (freely available from [27]), and leverages numerous tools included with CloudBioLinux: sequencing alignment is configurable from options such as Bowtie, BWA and Novoalign; BAM alignment processing takes advantage of the Picard toolkit; variant calling uses GATK; quality assessment utilizes FastQC. The CloudMan server [28] that is also integrated with CloudBioLinux allows parallelization of each of these tasks to reduce execution time, through distribution of the computation across nodes of a Sun Grid Engine (SGE) cluster. The cluster size is specified by the user and is automatically assembled by CloudMan, by instantiating and connecting on a cloud-based SGE grid a number of Cloud BioLinux VMs.

We have successfully completed this data analysis pipeline with a runtime in the order of a few hours, using approximately 700,000 paired-end Illumina HiSeq reads on a cluster of large-memory (m1.xlarge) instances on Amazon EC2. Measurements of performance and costs for different cluster setups is beyond the scope of the current manuscript, as our focus is to present the details of the Cloud BioLinux framework that enables development of bioinformatics infrastructures on the cloud. Several studies have gathered data on cloud computing performance for scientific applications (for example, [29]), while we also plan a follow up study presenting performance details of Cloud BioLinux running a set of complex bioinformatic data analysis pipelines.

## Discussion

Virtual Machines (VMs) that run on cloud computing platforms are an alternative to in-house informatics infrastructures for bioinformatic data analysis, requiring minimal set-up and no up-front hardware costs. Renting servers on the cloud can work as a better model for smaller research laboratories, where the cost for hardware and data center maintenance, cannot be justified to support only a few experiments. Using VMs allows for snapshots of the computing server to be taken, including the operating system and software, input data files configured settings and analysis results. The VM snapshots can be shared among collaborating researchers using a commercial cloud platform such as Amazon EC2, open source clouds including Eucalyptus or OpenStack, or desktop virtualization software like VirtualBox. Snapshots are an ideal approach for reproducibility of *in-silico *analyses, given that bioinformatics research involves small but important configuration changes while working with the different tools and datasets. These include for example tuning algorithm parameters in software installations, or making *ad-hoc *modifications to software for specific data processing cases, which are otherwise difficult to capture and share among collaborators.

Bioinformatics projects that use cloud technologies for software distribution, include for example CloVR, Bioconductor, Qiime and GMOD. These efforts have produced VMs containing software for specific projects and user communities. In contrast, our project offers a full-featured VM including a large number of bioinformatics tools, and a developer's framework for creating, maintaining and distributing customized versions of Cloud BioLinux. The unique feature of Cloud BioLinux is that can be leveraged for producing such tailored, cloud-based solutions, and by offering this option we hope to encourage the community to standardize around a shared automated framework that allows creating customized VMs for bioinformatics with targeted software, data and configurations.

The Cloud BioLinux project to date has been largely based on the Amazon EC2 commercial cloud, but private and academic clouds have become commonplace for computing provision. We believe that this trend will continue forward, since in-house clouds overcome the shortcoming of EC2 when large datasets are involved, which relates to bandwidth costs for transferring data to and from the cloud storage. On the other hand, Eucalyptus and Openstack open-source cloud platforms can be readily deployed by an organization to run on internal servers.

Compared to traditional computing clusters, when researchers use in-house clouds they gain the advantage of running distinct VMs that each encapsulates an operating system, analysis software and data. With this approach, users receive distinct and pre-defined computational resources, since each initiates their own set of VM servers. This prevents over-utilization of the hardware by a single user, as it often happens within a traditional multi-user, single-server environment. By allocating an appropriate number of VM servers, researchers can scale computational resources as needed to perform different tasks within bioinformatics data pipelines. For example, when running computationally intensive tasks an increased amount of computational capacity can be allocated by initiating a large number of VM servers. The number of VM servers can be scaled down for pipeline stages involving less computationally intensive tasks, such as those of genome annotation data browsing. This elastic capacity property and on-demand scaling of the computational resources, is a unique feature of the VM based cloud computing. Last but not least, the separation of each VM from the rest of the computing environment, translates to more flexibility in testing unstable software or experimenting with new bioinformatics approaches, without the side-effect of affecting production systems.

While it is already possible to run Cloud BioLinux images on Eucalyptus private clouds, our future plans is to make this process seamless by providing additional automation scripts, documentation and a range of pre-built VMs specifically for the Eucalyptus and OpenStack cloud platforms. In addition, we intend to implement tools that streamline data upload, management and sharing within the cloud, including readily available access to the bioinformatics datasets already hosted as public service free of charge on Amazon EC2. We also aim to work more closely with the community of the Debian-Med project, which provides a Linux distribution with a range of biomedical computing software packages. Finally, while Hadoop/MapReduce [30] is already available with Cloud BioLinux, in the next version of our VM we plan to implement specialized scripts that allow end-users to easily provision Hadoop clusters on any cloud. This will facilitate running large-scale bioinformatics data processing pipelines similar to Crossbow [31], and parallel genome assemblers such as Cloudburst [32] or Contrail [33].

## Conclusions

Our overall goal is to provide a platform for the community to center around a single bioinformatics cloud computing distribution and to focus on the next challenges of providing data, documentation, and the development of scalable analysis pipelines. The software management framework we developed using scripts and configuration files based on the Fabric system, allows Cloud BioLinux to be easily reproduced and updated since the configuration files provide an exact record of how the VM was configured. Furthermore, our framework is available through a distributed source code repository, which makes it easy for developers to participate in the project and create derivative systems. Finally, the Fabric scripts and configuration files can be modified so that the software included in the VM is tailored to the data analysis needs of each researcher, while also allowing deployment on public and private clouds or desktop computers, as we have demonstrated with the ready-to-execute images for the Eucalyptus cloud and VirtualBox available on our project website.

### Availability and Requirements

**Project name: **Cloud BioLinux

**Project home page: **http://www.cloudbiolinux.org

**Operating system(s): **Linux, Windows, Mac OSX

**Programming language: **Python

**Other requirements: **FreeNX desktop client, cloud computing or virtualization layer

**License: **MIT Licence, including derivatives and customized Cloud BioLinux VMs

**Any restrictions to use by non-academics: **none

## Abbreviations

VM(s): Virtual Machine(s); APT Debian: Aptitude Package Manager; SSH: Secure Shell

## Competing interests

The authors declare that they have no competing interests.

## Authors' contributions

KK, TB and BC have developed, tested and implemented the Cloud BioLinux software framework and cloud Virtual Machines, in addition to developing the manuscript to its final version. BT developed the Cloud BioLinux documentation. MB, DF and KN participated in the study design and coordination and helped to draft the manuscript. All authors read and approved the final manuscript.

## Supplementary Material

Additional file 1 **Supplementary 1 Cloud BioLinux software documentation in the form of a mini, self-contained website**. Users need to download and uncompress the .zip file, and open through a web browser the "index.html" file available on the main directory. (ZIP 1823 kb). Click here for file

Additional file 2 **Supplementary 2**. Overall, the pipeline takes as input sequencing reads, converts them to standard Fastq format, aligning to a reference genome, doing SNP calling, and producing a summary PDF of results. Furthermore, it leverages the CloudMan server that is included in Cloud BioLinux VM for provisioning a Sun Grid Engine (SGE) cluster composed of multiple copies of the VM on the cloud, and the RabbitMQ distributed messaging queue for data coordination and exchange between the cluster nodes respectively (**a**.) sequence files for each lane are split based on the sample barcodes and the parts are aligned using Bowtie to the genome in parallel, by being submitted as separate SGE computational tasks across the cluster nodes. The pipeline then sorts and merges BAM alignment files from multiple lanes if sequences have the same barcode, producing a single representative BAM file for each barcoded sub-sample. At this step, RabbitMQ enables message exchange between the cluster nodes in order to identify BAM sequence alignment sets with the same barcode on different nodes, and the pipeline scripts transfer and merge those sets on a single node (**b**.) the second phase parallelizes the processing of each alignment file with read quality assessment, variant calling and visualization, using an identical approach based on SGE parallel computation across cluster nodes, with the software tools performing each of these steps being respectively Fastqc and Picard, GATK and BigWig. (JPEG 160 kb). Click here for file
