# Supplementary material for: Cloud BioLinux: pre-configured and on-demand bioinformatics computing for the genomics community
Source: BMC Bioinformatics. 2012 Mar 19;13:42. doi: 10.1186/1471-2105-13-42 (PMC3372431; doi:10.1186/1471-2105-13-42)
Supplement: Additional file 1 — Supplementary 1 Cloud BioLinux software documentation in the form of a mini, self-contained website. Users need to download and uncompress the .zip file, and open through a web browser the "index.html" file available on the main directory. (ZIP 1823 kb). [file 1471-2105-13-42-S1.ZIP › Cloud-BioLinux-Package-Documentation/docs/clique.html]

Bio-Linux Software Documentation Pages

Back to search form

## clique

|  |  |
| --- | --- |
| Name | clique |
| Description | **clique** is part of the PHYLIP package  The following text is `Copyright 1986-2004` by the University of Washington. Written by Joseph Felsenstein.  This program uses the compatibility method for unrooted two-state characters to obtain the largest cliques of characters and the trees which they suggest. This approach originated in the work of Le Quesne (1969), though the algorithms were not precisely specified until the later work of Estabrook, Johnson, and McMorris (1976a, 1976b). These authors proved the theorem that a group of two-state characters which were pairwise compatible would be jointly compatible.  This program uses an algorithm inspired by the Kent Fiala - George Estabrook program CLINCH, though closer in detail to the algorithm of Bron and Kerbosch (1973). I am indebted to Kent Fiala for pointing out that paper to me, and to David Penny for decribing to me his branch-and-bound approach to finding largest cliques, from which I have also borrowed. I am particularly grateful to Kent Fiala for catching a bug in versions 2.0 and 2.1 which resulted in those versions failing to find all of the cliques which they should. The program computes a compatibility matrix for the characters, then uses a recursive procedure to examine all possible cliques of characters.  After one pass through all possible cliques, the program knows the size of the largest clique, and during a second pass it prints out the cliques of the right size. It also, along with each clique, prints out a the tree suggested by that clique.  **References:**  Felsenstein, J. 1993. PHYLIP (Phylogeny Inference Package) version 3.5c. Distributed by the author. Department of Genetics, University of Washington, Seattle.    Felsenstein, J. 1989. PHYLIP -- Phylogeny Inference Package (Version 3.2). Cladistics 5: 164-166. |
| Homepage | http://evolution.genetics.washington.edu/phylip.html |
| Remote Documentation | http://evolution.genetics.washington.edu/phylip/doc/clique.html |
