# Supplementary material for: Cloud BioLinux: pre-configured and on-demand bioinformatics computing for the genomics community
Source: BMC Bioinformatics. 2012 Mar 19;13:42. doi: 10.1186/1471-2105-13-42 (PMC3372431; doi:10.1186/1471-2105-13-42)
Supplement: Additional file 1 — Supplementary 1 Cloud BioLinux software documentation in the form of a mini, self-contained website. Users need to download and uncompress the .zip file, and open through a web browser the "index.html" file available on the main directory. (ZIP 1823 kb). [file 1471-2105-13-42-S1.ZIP › Cloud-BioLinux-Package-Documentation/docs/factor.html]

Bio-Linux Software Documentation Pages

Back to search form

## factor

|  |  |
| --- | --- |
| Name | factor |
| Description | **factor** is a part of the PHYLIP packageOn Bio-Linux 6.0, you can call this program by using either of the commands below:  `factor_phylip` `phylip factor`  Copyright 1986-2004 by The University of Washington. Written by Christopher Meacham and Joseph Felsenstein. Permission is granted to copy this document provided that no fee is charged for it and that this copyright notice is not removed.  This program factors a data set that contains multistate characters, creating a data set consisting entirely of binary (0,1) characters that, in turn, can be used as input to any of the other discrete character programs in this package, except for PARS. Besides this primary function, FACTOR also provides an easy way of deleting characters from a data set. The input format for FACTOR is very similar to the input format for the other discrete character programs except for the addition of character-state tree descriptions.  Note that this program has no way of converting an unordered multistate character into binary characters. Fortunately, PARS has joined the package, and it enables unordered multistate characters, in which any state can change to any other in one step, to be analyzed with parsimony.  FACTOR is really for a different case, that in which there are multiple states related on a character state tree, which specifies for each state which other states it can change to. That graph of states is assumed to be a tree, with no loops in it.  **References:**  Felsenstein, J. 1993. PHYLIP (Phylogeny Inference Package) version 3.5c. Distributed by the author. Department of Genetics, University of Washington, Seattle.    Felsenstein, J. 1989. PHYLIP -- Phylogeny Inference Package (Version 3.2). Cladistics 5: 164-166. |
| Homepage | http://evolution.genetics.washington.edu/phylip.html |
| Remote Documentation | http://evolution.genetics.washington.edu/phylip/doc/factor.html |
