# Supplementary material for: Cloud BioLinux: pre-configured and on-demand bioinformatics computing for the genomics community
Source: BMC Bioinformatics. 2012 Mar 19;13:42. doi: 10.1186/1471-2105-13-42 (PMC3372431; doi:10.1186/1471-2105-13-42)
Supplement: Additional file 1 — Supplementary 1 Cloud BioLinux software documentation in the form of a mini, self-contained website. Users need to download and uncompress the .zip file, and open through a web browser the "index.html" file available on the main directory. (ZIP 1823 kb). [file 1471-2105-13-42-S1.ZIP › Cloud-BioLinux-Package-Documentation/docs/infobase.html]

Bio-Linux Software Documentation Pages

Back to search form

## infobase

|  |  |
| --- | --- |
| Name | infobase |
| Description | **infobase:** reports the defined properties for all known bases, or for any bases specified by the user.  The sample output file in the official documentation is probably the best way to get an idea what infobase does.    **infobase -h** for basic programme options    **infobase -h -v** for further programme options     **tfm infobase**  for full program informations |
| Homepage | http://emboss.sourceforge.net |
| Remote Documentation | http://emboss.sourceforge.net/apps/release/6.3/emboss/apps/infobase.html |

EMBOSS documentation for infobase
