# Supplementary material for: Cloud BioLinux: pre-configured and on-demand bioinformatics computing for the genomics community
Source: BMC Bioinformatics. 2012 Mar 19;13:42. doi: 10.1186/1471-2105-13-42 (PMC3372431; doi:10.1186/1471-2105-13-42)
Supplement: Additional file 1 — Supplementary 1 Cloud BioLinux software documentation in the form of a mini, self-contained website. Users need to download and uncompress the .zip file, and open through a web browser the "index.html" file available on the main directory. (ZIP 1823 kb). [file 1471-2105-13-42-S1.ZIP › Cloud-BioLinux-Package-Documentation/docs/mview.html]

Bio-Linux Software Documentation Pages

Back to search form

## mview

|  |  |
| --- | --- |
| Name | mview |
| Description | **Mview** is a Blast reports post-processing tool.  **MView** is a tool for converting the results of a sequence database search (BLAST, FASTA, etc.) into the form of a coloured multiple alignment of hits stacked against the query. Alternatively, an existing multiple alignment (MSF, PIR, CLUSTAL, etc.) can be processed.  In either case, the output is simply HTML, so the result is platform independent and does not require a separate application or applet to be loaded. **MView** is NOT a multiple alignment program, nor is it a general purpose alignment editor.  **References:**  Brown, N.P., Leroy C., Sander C. (1998). MView: A Web compatible database search or multiple alignment viewer. Bioinformatics. 14(4):380-381. |
| Homepage | ftp://mathbio.nimr.mrc.ac.uk/pub/nbrown/MView/ |
| Remote Documentation | ftp://mathbio.nimr.mrc.ac.uk/pub/nbrown/MView/html/index.html |
