# Supplementary material for: Cloud BioLinux: pre-configured and on-demand bioinformatics computing for the genomics community
Source: BMC Bioinformatics. 2012 Mar 19;13:42. doi: 10.1186/1471-2105-13-42 (PMC3372431; doi:10.1186/1471-2105-13-42)
Supplement: Additional file 1 — Supplementary 1 Cloud BioLinux software documentation in the form of a mini, self-contained website. Users need to download and uncompress the .zip file, and open through a web browser the "index.html" file available on the main directory. (ZIP 1823 kb). [file 1471-2105-13-42-S1.ZIP › Cloud-BioLinux-Package-Documentation/docs/sequin.html]

Bio-Linux Software Documentation Pages

Back to search form

## sequin

|  |  |
| --- | --- |
| Name | sequin |
| Description | **sequin** is a stand-alone software tool developed by the National Center for Biotechnology Information (NCBI) for submitting and updating entries to the GenBank, EMBL, or DDBJ sequence databases. |
| Homepage | http://www.ncbi.nlm.nih.gov/Sequin/index.html |
| Remote Documentation | http://www.ncbi.nlm.nih.gov/Sequin/index.html   http://www.ncbi.nlm.nih.gov/Sequin/QuickGuide/sequin.htm |

Sequin is a stand-alone software tool developed by the National Center for
Biotechnology Information (NCBI) for submitting and updating entries to the GenBank, EMBL, or DDBJ sequence da
tabases.
