# Supplementary material for: Cloud BioLinux: pre-configured and on-demand bioinformatics computing for the genomics community
Source: BMC Bioinformatics. 2012 Mar 19;13:42. doi: 10.1186/1471-2105-13-42 (PMC3372431; doi:10.1186/1471-2105-13-42)
Supplement: Additional file 1 — Supplementary 1 Cloud BioLinux software documentation in the form of a mini, self-contained website. Users need to download and uncompress the .zip file, and open through a web browser the "index.html" file available on the main directory. (ZIP 1823 kb). [file 1471-2105-13-42-S1.ZIP › Cloud-BioLinux-Package-Documentation/docs/prfx.html]

Bio-Linux Software Documentation Pages

Back to search form

## prfx

|  |  |
| --- | --- |
| Name | prfx |
| Description | **prfx** is part of the fasta3 package. FASTA contains many programs for searching DNA and protein databases for evaluating statistical significance from randomly shuffled sequences.  **prfx** is used to evaluate the significance of a translated-DNA:protein sequence similarity score by comparing two sequences and calculating optimal similarity scores, and then repeatedly shuffling the second sequence, and calculating optimal similarity scores using the Smith-Waterman algorithm. An extreme value distribution is then fit to the shuffled-sequence scores. The characteristic parameters of the extreme value distribution are then used to estimate the probability that each of the unshuffled sequence scores would be obtained by chance in one sequence, or in a number of sequences equal to the number of shuffles. **prss** is a related program allowing evaluation of DNA:DNA and protein:protein matches.  **References:**  Pearson, W.R. Flexible sequence similarity searching with the FASTA3 program package. Methods Mol Biol. 2000;132:185-219 [Entrez]    Pearson, W.R. Empirical statistical estimates for sequence similarity searches. J Mol Biol. 1998 Feb 13;276(1):71-84 [Entrez]    Pearson WR, Wood T, Zhang Z, Miller W. Comparison of DNA sequences with protein sequences. Genomics. 1997 Nov 15;46(1):24-36. [Entrez] |
| Homepage | http://www.people.virginia.edu/~wrp/pearson.html |
| Remote Documentation | http://www.people.virginia.edu/~wrp/papers/ismb2000.pdf |
