# Supplementary material for: Cloud BioLinux: pre-configured and on-demand bioinformatics computing for the genomics community
Source: BMC Bioinformatics. 2012 Mar 19;13:42. doi: 10.1186/1471-2105-13-42 (PMC3372431; doi:10.1186/1471-2105-13-42)
Supplement: Additional file 1 — Supplementary 1 Cloud BioLinux software documentation in the form of a mini, self-contained website. Users need to download and uncompress the .zip file, and open through a web browser the "index.html" file available on the main directory. (ZIP 1823 kb). [file 1471-2105-13-42-S1.ZIP › Cloud-BioLinux-Package-Documentation/docs/cdbfasta.html]

Bio-Linux Software Documentation Pages

Back to search form

## cdbfasta

|  |  |
| --- | --- |
| Name | cdbfasta |
| Description | **cdbfasta** is one of two platform independent file-based tools released by G. Pertea at TIGR,w which are required for the qiime pipeline. cdbfasta creating indices for quick retrieval of any particular sequences from large multi-FASTA files. The partner program is called **cdbyank**, which is used to retrieve sequence entries from a file indexed using cdbfasta.  If you are interested in indexing fastafiles, we recommend you look at more commonly used alternatives that are available on Bio-Linux. One worth considering is dbifasta in the EMBOSS suite of tools. |
| Homepage | http://compbio.dfci.harvard.edu/tgi/software/ |
| Remote Documentation |  |
