# Supplementary material for: Cloud BioLinux: pre-configured and on-demand bioinformatics computing for the genomics community
Source: BMC Bioinformatics. 2012 Mar 19;13:42. doi: 10.1186/1471-2105-13-42 (PMC3372431; doi:10.1186/1471-2105-13-42)
Supplement: Additional file 1 — Supplementary 1 Cloud BioLinux software documentation in the form of a mini, self-contained website. Users need to download and uncompress the .zip file, and open through a web browser the "index.html" file available on the main directory. (ZIP 1823 kb). [file 1471-2105-13-42-S1.ZIP › Cloud-BioLinux-Package-Documentation/docs/qiime.html]

Bio-Linux Software Documentation Pages

Back to search form

## qiime

|  |  |
| --- | --- |
| Name | qiime |
| Description | **Qiime** is a pipeline for performing microbial community analysis. Qiime makes use of many third party tools; all of the *required* third party software is installed as dependencies of the bio-linux-qiime package.  **What the Bio-Linux Qiime package does not include:**   - **Required:**The bio-linux-qiime package does **not** come with the greengenes data files required to run some elements of qiime. You will need to download the greengenes core set data file, and the greengenes lanemask files yourself. If you want these available system-wide, make sure to put them somewhere accessible to all users on the system. (If you are working on a standard Bio-Linux machine, `/home/db` will be readable to all your users by default.)- **Optional:** gsAssembler, aka Newbler, from Roche. You will need to install this yourself if you wish to make use of the sffinfo binary.   **What you need to do to run qiime on Bio-Linux:**   1. Download the greengenes files as detailed above.- Start up the qiime environment by typing `qiime` on the command line. You can then run qiime commands at the qiime prompt.   For an introduction to the possibilities of qiime, please refer to the qiime tutorials.  A standard QIIME analysis can take as input sequence data from one or more sequencing platforms, including Sanger, Roche/454, and Illumina GAIIx. An example of the pipeline process you might wish to run within qiime would be:   - perform library de-multiplexing and quality filtering- denoise with denoise.py- OTU and representative set picking with uclust, cdhit, mothur, BLAST, or other tools- taxonomy assignment with BLAST or the RDP classifier- sequence alignment with PyNAST, muscle, infernal, or other tools- phylogeny reconstruction with FastTree, raxml, clearcut, or other tools- alpha diversity and rarefaction, including visualization of results, using over 20 metrics including Phylogenetic Diversity, chao1, and observed species- beta diversity and rarefaction, including visualization of results, using over 25 metrics including weighted and unweighted UniFrac, Euclidean distance, and Bray-Curtis; summarization and visualization of taxonomic composition of samples using pie charts and histograms   Qiime has many other features in addition to those listed above. Please see the Qiime homepage for further information. QIIME is an open-source project, developed primarily in the Knight Lab at the University of Colorado at Boulder. |
| Homepage | http://qiime.sourceforge.net/ |
| Remote Documentation | http://qiime.sourceforge.net/documentation/index.html   http://qiime.sourceforge.net/tutorials/index.html |
