# Supplementary material for: Cloud BioLinux: pre-configured and on-demand bioinformatics computing for the genomics community
Source: BMC Bioinformatics. 2012 Mar 19;13:42. doi: 10.1186/1471-2105-13-42 (PMC3372431; doi:10.1186/1471-2105-13-42)
Supplement: Additional file 1 — Supplementary 1 Cloud BioLinux software documentation in the form of a mini, self-contained website. Users need to download and uncompress the .zip file, and open through a web browser the "index.html" file available on the main directory. (ZIP 1823 kb). [file 1471-2105-13-42-S1.ZIP › Cloud-BioLinux-Package-Documentation/docs/cdbyank.html]

Bio-Linux Software Documentation Pages

Back to search form

## cdbyank

|  |  |
| --- | --- |
| Name | cdbyank |
| Description | **cdbyank** is a program used to retrieve records from an index file created using its sister program cdbfasta.  cdbyank and cdbfasta are programs released by G. Pertea of TIGR, and are included on Bio-Linux primarily as part of the Qiime pipeline for processing 16S sequences.  If you are interested in sequence indexing and retrieval tools, we recommend you consider more commonly used alternatives such as those offered within the EMBOSS suite. For indexing, try dbifasta. For sequence retrieval, try seqret. |
| Homepage | http://compbio.dfci.harvard.edu/tgi/software/ |
| Remote Documentation |  |
