# Supplementary material for: Cloud BioLinux: pre-configured and on-demand bioinformatics computing for the genomics community
Source: BMC Bioinformatics. 2012 Mar 19;13:42. doi: 10.1186/1471-2105-13-42 (PMC3372431; doi:10.1186/1471-2105-13-42)
Supplement: Additional file 1 — Supplementary 1 Cloud BioLinux software documentation in the form of a mini, self-contained website. Users need to download and uncompress the .zip file, and open through a web browser the "index.html" file available on the main directory. (ZIP 1823 kb). [file 1471-2105-13-42-S1.ZIP › Cloud-BioLinux-Package-Documentation/docs/promer.html]

Bio-Linux Software Documentation Pages

  
  
  


Back to search form

### promer

|  |  |
| --- | --- |
| Name | promer |
| Description | promer is a part of the MUMmer package, for the rapid alignment of very large DNA and amino acid sequences.   PROmer is a Perl script pipeline for the alignment of multiple somewhat divergent nucleotide sequences. It works exactly like NUCmer, but with a small twist. Before any of the exact matching takes place, the input sequences are translated in all six amino acid reading frames. This allows PROmer to identify regions of conserved protein sequences that may not be conserved on the DNA level and thus gives it a higher sensitivity than NUCmer. Note however, this increase in sensitivity will result in huge amounts of output for highly similar sequences, therefore it is recommended that PROmer only be used when the input sequences are too divergent to produce a reasonable amount of NUCmer output. As with NUCmer, it is recommended to mask the input sequences to avoid the alignment of uninteresting sequence, or to change the uniqueness constraints (see the PROmer section) to reduce the number of repeat induced alignments.      **References**  Delcher AL, Kasif S, Fleischmann RD, Peterson J, White O, Salzberg SL: Alignment of whole genomes, Nucleic Acids Res. 1999 Jun 1;27(11):2369-76.[Entrez]   Delcher AL, Phillippy A, Carlton J, Salzberg SL: Fast algorithms for large-scale genome alignment and comparison, Nucleic Acids Res. 2002 Jun 1;30(11):2478-83.[Entrez]   Kurtz S, Phillippy A, Delcher AL, Smoot M, Shumway M, Antonescu C, Salzberg SL: Versatile and open software for comparing large genomes, Genome Biol. 2004;5(2):R12. Epub 2004 Jan 30.[Entrez] |
| Homepage | http://www.tigr.org/software/mummer/ |
| Remote Documentation | http://www.tigr.org/software/mummer/manual/ |
