# Supplementary material for: Cloud BioLinux: pre-configured and on-demand bioinformatics computing for the genomics community
Source: BMC Bioinformatics. 2012 Mar 19;13:42. doi: 10.1186/1471-2105-13-42 (PMC3372431; doi:10.1186/1471-2105-13-42)
Supplement: Additional file 1 — Supplementary 1 Cloud BioLinux software documentation in the form of a mini, self-contained website. Users need to download and uncompress the .zip file, and open through a web browser the "index.html" file available on the main directory. (ZIP 1823 kb). [file 1471-2105-13-42-S1.ZIP › Cloud-BioLinux-Package-Documentation/docs/ssake.html]

Bio-Linux Software Documentation Pages

Back to search form

## ssake

|  |  |
| --- | --- |
| Name | ssake |
| Description | **ssake** - or The Short Sequence Assembly by K-mer search and 3' read Extension - assembles millions of short nucleotide sequences. It progressively searching for perfect 3'-most k-mers using a DNA prefix tree. ssake cycles through short sequence reads stored in a hash table and progressively searches through a prefix tree for the longest possible identical overlap between any two sequences.  **Note:**The ssake documentation strongly recommends using the **tqs** script for quality trimming reads before assembly. You can find out more by referring to the man page: aafter installing ssake, type: `man ssake` `man tqs` or by referring to the remote documentation. The ssake package is prepared by Debian-med and can be installed on Bio-Linux by typing: `sudo apt-get install ssake` For further details, please see the remote documentation.  Other tools available on Bio-Linux for handling data from new sequencing technologies are described in the bioinformatics documentation on the NEBC website. |
| Homepage | http://www.bcgsc.ca/platform/bioinfo/software/ssake |
| Remote Documentation | http://www.bcgsc.ca/platform/bioinfo/software/ssake      http://www.bcgsc.ca/platform/Members/rwarren/ssake08.pdf |
