# Supplementary material for: Cloud BioLinux: pre-configured and on-demand bioinformatics computing for the genomics community
Source: BMC Bioinformatics. 2012 Mar 19;13:42. doi: 10.1186/1471-2105-13-42 (PMC3372431; doi:10.1186/1471-2105-13-42)
Supplement: Additional file 1 — Supplementary 1 Cloud BioLinux software documentation in the form of a mini, self-contained website. Users need to download and uncompress the .zip file, and open through a web browser the "index.html" file available on the main directory. (ZIP 1823 kb). [file 1471-2105-13-42-S1.ZIP › Cloud-BioLinux-Package-Documentation/docs/gendist.html]

Bio-Linux Software Documentation Pages

Back to search form

## gendist

|  |  |
| --- | --- |
| Name | gendist |
| Description | **gendist** is a part of the PHYLIP package  Copyright 1986-2004 by the University of Washington. Written by Joseph Felsenstein. Permission is granted to copy this document provided that no fee is charged for it and that this copyright notice is not removed.  This program computes any one of three measures of genetic distance from a set of gene frequencies in different populations (or species). The three are Nei's genetic distance (Nei, 1972), Cavalli-Sforza's chord measure (Cavalli- Sforza and Edwards, 1967) and Reynolds, Weir, and Cockerham's (1983) genetic distance. These are written to an output file in a format that can be read by the distance matrix phylogeny programs FITCH and KITSCH.  **References**  Felsenstein, J. 1993. PHYLIP (Phylogeny Inference Package) version 3.5c. Distributed by the author. Department of Genetics, University of Washington, Seattle.    Felsenstein, J. 1989. PHYLIP -- Phylogeny Inference Package (Version 3.2). Cladistics 5: 164-166. |
| Homepage | http://evolution.genetics.washington.edu/phylip.html |
| Remote Documentation | http://evolution.genetics.washington.edu/phylip/doc/gendist.html |
