# Supplementary material for: Cloud BioLinux: pre-configured and on-demand bioinformatics computing for the genomics community
Source: BMC Bioinformatics. 2012 Mar 19;13:42. doi: 10.1186/1471-2105-13-42 (PMC3372431; doi:10.1186/1471-2105-13-42)
Supplement: Additional file 1 — Supplementary 1 Cloud BioLinux software documentation in the form of a mini, self-contained website. Users need to download and uncompress the .zip file, and open through a web browser the "index.html" file available on the main directory. (ZIP 1823 kb). [file 1471-2105-13-42-S1.ZIP › Cloud-BioLinux-Package-Documentation/docs/cross_match.html]

Bio-Linux Software Documentation Pages

Back to search form

## cross\_match

|  |  |
| --- | --- |
| Name | cross\_match |
| Description | **Cross\_match** is a general purpose application for comparing any two DNA sequence sets. For example, it can be used to compare a set of reads to a set of vector sequences and produce vector-masked versions of the reads, a set of cDNA sequences to a set of cosmids, contig sequences found by two alternative assembly procedures (for example, phrap and xbap) to each other, or phrap contigs to the final edited cosmid sequence. It is slower but more sensitive than BLAST.  **Cross\_match** is required and/or recommended by some packages available on Bio-Linux such as trace2dbest.  **Cross\_match** is ***licensed software*** and thus is ***not*** installed by default on Bio-Linux. It is free to academic researchers and comes as part of the phrap package, but you must obtain a license to get the software. Further information on how to obtain phrap/cross\_match/swat is available on the official website for phred, phrap and consed. |
| Homepage | http://www.phrap.org |
| Remote Documentation | http://www.phrap.org/phredphrapconsed.html#block\_phrap   http://www.phrap.org/phredphrap/general.html |
