# Supplementary material for: Cloud BioLinux: pre-configured and on-demand bioinformatics computing for the genomics community
Source: BMC Bioinformatics. 2012 Mar 19;13:42. doi: 10.1186/1471-2105-13-42 (PMC3372431; doi:10.1186/1471-2105-13-42)
Supplement: Additional file 1 — Supplementary 1 Cloud BioLinux software documentation in the form of a mini, self-contained website. Users need to download and uncompress the .zip file, and open through a web browser the "index.html" file available on the main directory. (ZIP 1823 kb). [file 1471-2105-13-42-S1.ZIP › Cloud-BioLinux-Package-Documentation/docs/glam2.html]

Bio-Linux Software Documentation Pages

Back to search form

## glam2

|  |  |
| --- | --- |
| Name | glam2 |
| Description | glam2 is a software package for finding motifs in sequences, typically amino-acid or nucleotide sequences. The main innovation of glam2 is that it allows insertions and deletions in motifs. The glam2 package is prepared by Debian-Med and can be installed in Bio-Linux by typing: `sudo apt-get install glam2` Glam2 can also be run as part of the MEME suite on the web.  For further information about Glam2 please see the remote documentation links |
| Homepage | http://bioinformatics.org.au/glam2/ |
| Remote Documentation | http://bioinformatics.org.au/glam2/doc/ |
