# Supplementary material for: Cloud BioLinux: pre-configured and on-demand bioinformatics computing for the genomics community
Source: BMC Bioinformatics. 2012 Mar 19;13:42. doi: 10.1186/1471-2105-13-42 (PMC3372431; doi:10.1186/1471-2105-13-42)
Supplement: Additional file 1 — Supplementary 1 Cloud BioLinux software documentation in the form of a mini, self-contained website. Users need to download and uncompress the .zip file, and open through a web browser the "index.html" file available on the main directory. (ZIP 1823 kb). [file 1471-2105-13-42-S1.ZIP › Cloud-BioLinux-Package-Documentation/docs/generate.html]

Bio-Linux Software Documentation Pages

Back to search form

## generate

|  |  |
| --- | --- |
| Name | generate |
| Description | **generate** is a part of the Glimmer package, for finding genes in microbial DNA, especially the genomes of bacteria, archaea, and viruses.  This program reads an interpolated context model and then generates an artificial genome using the model to create the genes and iid sequence for the intergenic regions.  Command-line options specify the genome length, gc composition, and gene-length, intergenic length ranges.  **References:**  Salzberg SL, Delcher AL, Kasif S, White O: Microbial gene identification using interpolated Markov models. Nucleic Acids Res. 1998 Jan 15;26(2):544-8. [Entrez]    Delcher AL, Harmon D, Kasif S, White O, Salzberg SL: Improved microbial gene identification with GLIMMER. Nucleic Acids Res. 1999 Dec 1;27(23):4636-41. [Entrez] |
| Homepage | http://www.tigr.org/software/glimmer/ |
| Remote Documentation | http://www.tigr.org/software/glimmer/glimmer.readme |
