# Supplementary material for: Cloud BioLinux: pre-configured and on-demand bioinformatics computing for the genomics community
Source: BMC Bioinformatics. 2012 Mar 19;13:42. doi: 10.1186/1471-2105-13-42 (PMC3372431; doi:10.1186/1471-2105-13-42)
Supplement: Additional file 1 — Supplementary 1 Cloud BioLinux software documentation in the form of a mini, self-contained website. Users need to download and uncompress the .zip file, and open through a web browser the "index.html" file available on the main directory. (ZIP 1823 kb). [file 1471-2105-13-42-S1.ZIP › Cloud-BioLinux-Package-Documentation/docs/blixem.html]

Bio-Linux Software Documentation Pages

Back to search form

## blixem

|  |  |
| --- | --- |
| Name | blixem |
| Description | **Blixem**, which stands for BLast matches In an X-windows Embedded Multiple alignment, is an interactive browser of pairwise Blast matches that have been stacked up in a master-slave multiple alignment. It is thus not a 'true' multiple alignment, such as produced by e.g. Clustalw, but a 'one-to-many' alignment.  The command line usage for blixem is in the format **blixem [options] {sequencefile} {datafile} [X options]**  Both "sequencefile" and "datafile" can be substituted by "-" for reading from stdin (pipe). If "sequencefile" is piped, the first line should contain the sequence name and the second the sequence itself.  Options can be:  -s {mode} Sorting mode at startup.   - s = by Score- i = by Identity   - n = by Name   - p = by Position  -I Inverted sorting order  -b Don't start with Big Picture  -S {#} Start display at position #.   -F {file} Read in query sequence and data from  (replaces sequencefile).   -h Help and more options.  Some X options can be:  -acefont {font} Main font.   -font {font} Menu font.  To make the datafile from blast output, run MSPcrunch with option -q.  Related Resources from Sonhammer group see: here **References:**  Sonnhammer, ELL and Durbin, R (1994). A workbench for Large Scale Sequence Homology Analysis. Comput. Applic. Biosci, 10:301-307. [ENTREZ]    Sonnhammer, ELL and Durbin, R (1994). An expert system for processing sequence homology data. ISMB 2:363-368 [ENTREZ] |
| Homepage | http://www.cgr.ki.se/cgr/groups/sonnhammer/Blixem.html |
| Remote Documentation | http://www.cgr.ki.se/cgr/groups/sonnhammer/Blixem.html |
