# Supplementary material for: Cloud BioLinux: pre-configured and on-demand bioinformatics computing for the genomics community
Source: BMC Bioinformatics. 2012 Mar 19;13:42. doi: 10.1186/1471-2105-13-42 (PMC3372431; doi:10.1186/1471-2105-13-42)
Supplement: Additional file 1 — Supplementary 1 Cloud BioLinux software documentation in the form of a mini, self-contained website. Users need to download and uncompress the .zip file, and open through a web browser the "index.html" file available on the main directory. (ZIP 1823 kb). [file 1471-2105-13-42-S1.ZIP › Cloud-BioLinux-Package-Documentation/docs/blastn.html]

Bio-Linux Software Documentation Pages

Back to search form

## blastn

|  |  |
| --- | --- |
| Name | blastn |
| Description | blastn is part of the new blast+ package from the NCBI.  blastn searches a nucleotide database using a nucleotide query.  Help on the options available can be found by typing `blastn -help`  blastn understands the concept of tasks. The following tasks can be requested using the `-task` option and giving one of the following as the argument:  - **blastn**: Traditional BLASTN requiring an exact match of 11 - **blastn-short**: BLASTN program optimized for sequences shorter than 50 bases - **megablast**: Traditional megablast used to find very similar (e.g., intraspecies or closely related species) sequencesdc-megablast - **Discontiguous megablast used to find more distant (e.g., interspecies) sequences**  Indexed searches for megablast are available and are faster than regular megablast. The application to generate the database indices is called makembindex. |
| Homepage | http://www.ncbi.nlm.nih.gov/bookshelf/br.fcgi?book=helpblast∂=CmdLineAppsManual |
| Remote Documentation | http://www.ncbi.nlm.nih.gov/bookshelf/br.fcgi?book=helpblast∂=CmdLineAppsManual    http://www.ncbi.nlm.nih.gov/bookshelf/br.fc |

User manual for blast+ (blastn) programs
