# Supplementary material for: Cloud BioLinux: pre-configured and on-demand bioinformatics computing for the genomics community
Source: BMC Bioinformatics. 2012 Mar 19;13:42. doi: 10.1186/1471-2105-13-42 (PMC3372431; doi:10.1186/1471-2105-13-42)
Supplement: Additional file 1 — Supplementary 1 Cloud BioLinux software documentation in the form of a mini, self-contained website. Users need to download and uncompress the .zip file, and open through a web browser the "index.html" file available on the main directory. (ZIP 1823 kb). [file 1471-2105-13-42-S1.ZIP › Cloud-BioLinux-Package-Documentation/docs/pars.html]

Bio-Linux Software Documentation Pages

Back to search form

## pars

|  |  |
| --- | --- |
| Name | pars |
| Description | **pars** is part of the PHYLIP program.  Copyright 1986-2004 by the University of Washington. Written by Joseph Felsenstein. Permission is granted to copy this document provided that no fee is charged for it and that this copyright notice is not removed.  **pars** is a general parsimony program which carries out the Wagner parsimony method with multiple states. Wagner parsimony allows changes among all states. The criterion is to find the tree which requires the minimum number of changes. The Wagner method was originated by Eck and Dayhoff (1966) and by Kluge and Farris (1969). |
| Homepage | http://evolution.genetics.washington.edu/phylip.html |
| Remote Documentation | http://evolution.genetics.washington.edu/phylip/doc/pars.html |
