# Supplementary material for: Cloud BioLinux: pre-configured and on-demand bioinformatics computing for the genomics community
Source: BMC Bioinformatics. 2012 Mar 19;13:42. doi: 10.1186/1471-2105-13-42 (PMC3372431; doi:10.1186/1471-2105-13-42)
Supplement: Additional file 1 — Supplementary 1 Cloud BioLinux software documentation in the form of a mini, self-contained website. Users need to download and uncompress the .zip file, and open through a web browser the "index.html" file available on the main directory. (ZIP 1823 kb). [file 1471-2105-13-42-S1.ZIP › Cloud-BioLinux-Package-Documentation/docs/neighbor.html]

Bio-Linux Software Documentation Pages

Back to search form

## neighbor

|  |  |
| --- | --- |
| Name | neighbor |
| Description | **neighbor** is part of the PHYLIP package.  Copyright 1991-2004 by the University of Washington. Written by Joseph Felsenstein. Permission is granted to copy this document provided that no fee is charged for it and that this copyright notice is not removed.  This program implements the Neighbor-Joining method of Saitou and Nei (1987) and the UPGMA method of clustering. The program was written by Mary Kuhner and Jon Yamato, using some code from program FITCH. An important part of the code was translated from FORTRAN code from the neighbor-joining program written by Naruya Saitou and by Li Jin, and is used with the kind permission of Drs. Saitou and Jin.  **neighbor** constructs a tree by successive clustering of lineages, setting branch lengths as the lineages join. The tree is not rearranged thereafter. The tree does not assume an evolutionary clock, so that it is in effect an unrooted tree. It should be somewhat similar to the tree obtained by FITCH. The program cannot evaluate a User tree, nor can it prevent branch lengths from becoming negative. However the algorithm is far faster than FITCH or KITSCH. This will make it particularly effective in their place for large studies or for bootstrap or jackknife resampling studies which require runs on multiple data sets.  **References:**  Felsenstein, J. 1993. PHYLIP (Phylogeny Inference Package) version 3.5c. Distributed by the author. Department of Genetics, University of Washington, Seattle.    Felsenstein, J. 1989. PHYLIP -- Phylogeny Inference Package (Version 3.2). Cladistics 5: 164-166. |
| Homepage | http://evolution.genetics.washington.edu/phylip.html |
| Remote Documentation | http://evolution.genetics.washington.edu/phylip/doc/neighbor.html |
