# Supplementary material for: Cloud BioLinux: pre-configured and on-demand bioinformatics computing for the genomics community
Source: BMC Bioinformatics. 2012 Mar 19;13:42. doi: 10.1186/1471-2105-13-42 (PMC3372431; doi:10.1186/1471-2105-13-42)
Supplement: Additional file 1 — Supplementary 1 Cloud BioLinux software documentation in the form of a mini, self-contained website. Users need to download and uncompress the .zip file, and open through a web browser the "index.html" file available on the main directory. (ZIP 1823 kb). [file 1471-2105-13-42-S1.ZIP › Cloud-BioLinux-Package-Documentation/docs/tqs.html]

Bio-Linux Software Documentation Pages

Back to search form

## tqs

|  |  |
| --- | --- |
| Name | tqs |
| Description | **tqs** is part of the ssake package. It is a script for quality trimming data from the Illumina Platform, using user-defined thresholds. You can find out more by referring to the man page: after installing the ssake package, type: `man tqs` The ssake documentation states that running tqs on your Illumina data before proceeding with assembly is highly recommended.  Other tools available on Bio-Linux for handling data from new sequencing technologies are described in the bioinformatics documentation on the NEBC website. |
| Homepage | http://www.bcgsc.ca/platform/bioinfo/software/ssake |
| Remote Documentation |  |
