# Supplementary material for: Cloud BioLinux: pre-configured and on-demand bioinformatics computing for the genomics community
Source: BMC Bioinformatics. 2012 Mar 19;13:42. doi: 10.1186/1471-2105-13-42 (PMC3372431; doi:10.1186/1471-2105-13-42)
Supplement: Additional file 1 — Supplementary 1 Cloud BioLinux software documentation in the form of a mini, self-contained website. Users need to download and uncompress the .zip file, and open through a web browser the "index.html" file available on the main directory. (ZIP 1823 kb). [file 1471-2105-13-42-S1.ZIP › Cloud-BioLinux-Package-Documentation/docs/mira-3rd-party.html]

Bio-Linux Software Documentation Pages

Back to search form

## mira-3rd-party

|  |  |
| --- | --- |
| Name | mira-3rd-party |
| Description | **mira-3rd-party-scripts** consists of scripts distribruted by the creator of Mira, on behalf of others. These scripts are very useful for working with data from new sequencing technologies such as 454 or Solexa. The scripts in the bio-linux-mira-3rd-party package are:   - **454pairedEnd2caf.pl** - one of two scripts in this package to transform 454 paired end read data into common assembly format (caf) - **bin\_fasta\_on\_mid\_primers.pl** - bins fasta sequences into separate files based on mid primer sequences. Also strips the primer sequence. - **lucy2xml.pl** - convert files from lucy to xml format - **readpair2caf.pl** one of two scripts in this package to transform 454 paired end read data into common assembly format (caf) - **sff\_extract** - extract sequences from SFF files; also provides warnings if it appears that primers have not been clipped correctly and allows for various manipulations and output file formats.   To find out how to run these scripts, run the script name followed by **-h**. For example: `bin_fasta_on_mid_primers.pl -h` |
| Homepage | http://chevreux.org/mira\_downloads.html |
| Remote Documentation |  |
