# Supplementary material for: Cloud BioLinux: pre-configured and on-demand bioinformatics computing for the genomics community
Source: BMC Bioinformatics. 2012 Mar 19;13:42. doi: 10.1186/1471-2105-13-42 (PMC3372431; doi:10.1186/1471-2105-13-42)
Supplement: Additional file 1 — Supplementary 1 Cloud BioLinux software documentation in the form of a mini, self-contained website. Users need to download and uncompress the .zip file, and open through a web browser the "index.html" file available on the main directory. (ZIP 1823 kb). [file 1471-2105-13-42-S1.ZIP › Cloud-BioLinux-Package-Documentation/docs/rpsblast+.html]

Bio-Linux Software Documentation Pages

Back to search form

## rpsblast+

|  |  |
| --- | --- |
| Name | rpsblast+ |
| Description | rpsblast+ is part of the new blast+ package from the NCBI.  rpsblast+ searches a query sequence against a database of profiles, or score matrices, prepared by copymat, producing BLAST-like output.  Help on the options available can be found by typing `rpsblast+ -help` rpsblast+ is a name used in the bio-linux-blast+ package to avoid a naming conflict between the old and new rpsblast programs. **Please note** that on systems that are not Bio-Linux, you may have a program called rpsblast on your system. It may be from either the old blast software or the new blast+ software release; you will need to find out which.  If you type the command `rpsblast` on Bio-Linux, you will call the old rpsblast program. Please use `rpsblast+` instead. |
| Homepage | http://www.ncbi.nlm.nih.gov/bookshelf/br.fcgi?book=helpblast∂=CmdLineAppsManual |
| Remote Documentation | http://www.ncbi.nlm.nih.gov/bookshelf/br.fcgi?book=helpblast∂=CmdLineAppsManual      http://www.ncbi.nlm.nih.gov/bookshelf/br.fc |

User manual for blast+ (rpsblast+) programs
