# Supplementary material for: Cloud BioLinux: pre-configured and on-demand bioinformatics computing for the genomics community
Source: BMC Bioinformatics. 2012 Mar 19;13:42. doi: 10.1186/1471-2105-13-42 (PMC3372431; doi:10.1186/1471-2105-13-42)
Supplement: Additional file 1 — Supplementary 1 Cloud BioLinux software documentation in the form of a mini, self-contained website. Users need to download and uncompress the .zip file, and open through a web browser the "index.html" file available on the main directory. (ZIP 1823 kb). [file 1471-2105-13-42-S1.ZIP › Cloud-BioLinux-Package-Documentation/docs/run-mummer3.html]

Bio-Linux Software Documentation Pages

Back to search form

## run-mummer3

|  |  |
| --- | --- |
| Name | run-mummer3 |
| Description | **run-mummer3** is a part of the MUMmer package, for the rapid alignment of very large DNA and amino acid sequences.  **run-mummer3** is the simplest pipeline of the latest MUMmer3.0 programs. It runs the same matching and clustering algorithm as nucmer and promer, however it uses a different extension technique and does not perform the important pre- and post-processing steps of NUC/PROmer. Because of its simplistic form, run-mummer3 can only handle a single reference sequence, but like run-mummer1 its error-focused output makes it a handy tool for detecting SNPs and other small errors. The only major difference between run-mummer3 and run-mummer1 is the new version's ability to handle multiple query sequences and its tolerance of large rearrangements. This makes run-mummer3 well suited for error detection between highly similar sequences that may have large rearrangements, inversions etc.  Edit the script by adding the -D option to the combineMUMs command line to output a format designed for SNP identification.  **References:**  Delcher AL, Kasif S, Fleischmann RD, Peterson J, White O, Salzberg SL: Alignment of whole genomes, Nucleic Acids Res. 1999 Jun 1;27(11):2369-76.[Entrez]    Delcher AL, Phillippy A, Carlton J, Salzberg SL: Fast algorithms for large-scale genome alignment and comparison, Nucleic Acids Res. 2002 Jun 1;30(11):2478-83.[Entrez]    Kurtz S, Phillippy A, Delcher AL, Smoot M, Shumway M, Antonescu C, Salzberg SL: Versatile and open software for comparing large genomes, Genome Biol. 2004;5(2):R12. Epub 2004 Jan 30.[Entrez] |
| Homepage | http://www.tigr.org/software/mummer/ |
| Remote Documentation | http://www.tigr.org/software/mummer/manual/ |
