# Supplementary material for: Cloud BioLinux: pre-configured and on-demand bioinformatics computing for the genomics community
Source: BMC Bioinformatics. 2012 Mar 19;13:42. doi: 10.1186/1471-2105-13-42 (PMC3372431; doi:10.1186/1471-2105-13-42)
Supplement: Additional file 1 — Supplementary 1 Cloud BioLinux software documentation in the form of a mini, self-contained website. Users need to download and uncompress the .zip file, and open through a web browser the "index.html" file available on the main directory. (ZIP 1823 kb). [file 1471-2105-13-42-S1.ZIP › Cloud-BioLinux-Package-Documentation/docs/protpars.html]

Bio-Linux Software Documentation Pages

Back to search form

## protpars

|  |  |
| --- | --- |
| Name | protpars |
| Description | **protpars** is part of the PHYLIP package.  Copyright 1986-2004 by the University of Washington. Written by Joseph Felsenstein. Permission is granted to copy this document provided that no fee is charged for it and that this copyright notice is not removed.  This program infers an unrooted phylogeny from protein sequences, using a new method intermediate between the approaches of Eck and Dayhoff (1966) and Fitch (1971). Eck and Dayhoff (1966) allowed any amino acid to change to any other, and counted the number of such changes needed to evolve the protein sequences on each given phylogeny. This has the problem that it allows replacements which are not consistent with the genetic code, counting them equally with replacements that are consistent. Fitch, on the other hand, counted the minimum number of nucleotide substitutions that would be needed to achieve the given protein sequences. This counts silent changes equally with those that change the amino acid.  **References**  Felsenstein, J. 1993. PHYLIP (Phylogeny Inference Package) version 3.5c. Distributed by the author. Department of Genetics, University of Washington, Seattle.    Felsenstein, J. 1989. PHYLIP -- Phylogeny Inference Package (Version 3.2). Cladistics 5: 164-166. |
| Homepage | http://evolution.genetics.washington.edu/phylip.html |
| Remote Documentation | http://evolution.genetics.washington.edu/phylip/doc/protpars.html |
