# Supplementary material for: Cloud BioLinux: pre-configured and on-demand bioinformatics computing for the genomics community
Source: BMC Bioinformatics. 2012 Mar 19;13:42. doi: 10.1186/1471-2105-13-42 (PMC3372431; doi:10.1186/1471-2105-13-42)
Supplement: Additional file 1 — Supplementary 1 Cloud BioLinux software documentation in the form of a mini, self-contained website. Users need to download and uncompress the .zip file, and open through a web browser the "index.html" file available on the main directory. (ZIP 1823 kb). [file 1471-2105-13-42-S1.ZIP › Cloud-BioLinux-Package-Documentation/docs/cd-hit-est.html]

Bio-Linux Software Documentation Pages

Back to search form

## cd-hit-est

|  |  |
| --- | --- |
| Name | cd-hit-est |
| Description | **cd-hit-est** is part of a suite of programs designed to quickly group sequences.  cd-hit-est groups nucleotide sequences (without introns) into clusters that meet a user-defined similarity threshold.  Input is a fasta file of protein sequences. Output is a file of (non-redundant) representative sequences and a file listing the proteins in each cluster.  Other programs in this suite are:  - **mcd-hit** - a modified version of cd-hit, designed for sets of proteins of very different lengths. It uses a low clustering threshold. - **cd-hit-2d** - compares two protein data sets. It provides a list similar sequences in the two sets, and a list of sequnces in the second set that are not similar to sequences in the first set. - **cd-hit** - similar to cd-hit-est, but designed to group protein sequences. - **cd-hit-est-2d** - similar to cd-hit-2d but designed to compare two nucleotide datasets.  The scripts below use the output files (.clstr files) as input and generate reports.  - **plot\_len.pl** - generates a text file of distributions of clusters and sequences. - **clstr\_sort\_by.pl** - sorts clusters by length and number of sequences in the cluster. - **clstr\_sort\_prot\_by.pl** - sorts sequences within clusters by length and name. - **clstr\_merge.pl** - merge two or more .clstr files. - **clstr\_renumber.pl** - re-numbers clusters and sequences within clusters in a clstr file after merging (or other operations). - **clstr\_rev.pl** - combines a .clstr file with its parent .clstr file. - **make\_multi\_seq.pl** - reads a .clstr file and makes a fasta file for each cluster over a certain size. |
| Homepage | http://cd-hit.org |
| Remote Documentation | http://bioinformatics.oxfordjournals.org/cgi/reprint/22/13/1658      http://www.bioinformatics.org/cd-hit/cd-hit-user-guide.pdf |

cd-hit userguide
