# Supplementary material for: Cloud BioLinux: pre-configured and on-demand bioinformatics computing for the genomics community
Source: BMC Bioinformatics. 2012 Mar 19;13:42. doi: 10.1186/1471-2105-13-42 (PMC3372431; doi:10.1186/1471-2105-13-42)
Supplement: Additional file 1 — Supplementary 1 Cloud BioLinux software documentation in the form of a mini, self-contained website. Users need to download and uncompress the .zip file, and open through a web browser the "index.html" file available on the main directory. (ZIP 1823 kb). [file 1471-2105-13-42-S1.ZIP › Cloud-BioLinux-Package-Documentation/docs/restdist.html]

Bio-Linux Software Documentation Pages

Back to search form

## restdist

|  |  |
| --- | --- |
| Name | restdist |
| Description | **Restdist** is part of the PHYLIP package  Copyright 2000-2004 by the University of Washington. Written by Joseph Felsenstein. Permission is granted to copy this document provided that no fee is charged for it and that this copyright notice is not removed.  **Restdist** reads the same restriction sites format as RESTML and computes a restriction sites distance. It can also compute a restriction fragments distance. The original restriction fragments and restriction sites distance methods were introduced by Nei and Li (1979). Their original method for restriction fragments is also available in this program, although its default methods are my modifications of the original Nei and Li methods.  These two distances assume that the restriction sites are accidental byproducts of random change of nucleotide sequences. For my restriction sites distance the DNA sequences are assumed to be changing according to the Kimura 2-parameter model of DNA change (Kimura, 1980). The user can set the transition/transversion rate for the model. For my restriction fragments distance there is there is an implicit assumption of a Jukes-Cantor (1969) model of change, The user can also set the parameter of a correction for unequal rates of evolution between sites in the DNA sequences, using a Gamma distribution of rates among sites. The Jukes-Cantor model is also implicit in the restriction fragments distance of Nei and Li(1979). It does not allow us to correct for a Gamma distribution of rates among sites. |
| Homepage | http://evolution.genetics.washington.edu/phylip.html |
| Remote Documentation | http://evolution.genetics.washington.edu/phylip/doc/restdist.html |
