# Supplementary material for: Cloud BioLinux: pre-configured and on-demand bioinformatics computing for the genomics community
Source: BMC Bioinformatics. 2012 Mar 19;13:42. doi: 10.1186/1471-2105-13-42 (PMC3372431; doi:10.1186/1471-2105-13-42)
Supplement: Additional file 1 — Supplementary 1 Cloud BioLinux software documentation in the form of a mini, self-contained website. Users need to download and uncompress the .zip file, and open through a web browser the "index.html" file available on the main directory. (ZIP 1823 kb). [file 1471-2105-13-42-S1.ZIP › Cloud-BioLinux-Package-Documentation/docs/mothur.html]

Bio-Linux Software Documentation Pages

Back to search form

## mothur

|  |  |
| --- | --- |
| Name | mothur |
| Description | Mothur provides microbial ecologists with the functionality of dotur, sons, treeclimber, s-libshuff, unifrac and more.  Mothur is a project initiated by Dr. Patrick Schloss. It is a single piece of software that incorporates the functionality of dotur, sons, treeclimber, s-libshuff, uni frac and more. In essense, the goal of mothur is to provide a single resource to analyse molecular data used by microbial ecologists.  Functionality includes sequence processing, allowing you to go from Sanger sequences or pyrosequences to a distance matrix, carry out OTU-based approaches and hypothes is testing appropaches in considering the frequency distribution of sequences found in bins.  As of version 1.11 on Bio-Linux, mothur can run under mpi, which allows you to make use of multiple cores. To run mothur under mpi, you could use a command like: `mpirun -np 2 mothur` The above would run mothur making use of 2 cpu, assuming two are available.  To run commands under mpirun, you need to have mpd running on your system. So the full set of commands you might need to run, if mpd is not already set up and running, is below. (Replace the word secret with an actual, secret password.) `echo "MPD_SECRETWORD=secret"  ~/.mpd.conf  chmod 600 ~/.mpd.conf  mpd &  mpirun -np 2 mothur` |
| Homepage | http://www.mothur.org/ |
| Remote Documentation | http://www.mothur.org/wiki      http://www.mothur.org/forum/ |
