# Supplementary material for: Cloud BioLinux: pre-configured and on-demand bioinformatics computing for the genomics community
Source: BMC Bioinformatics. 2012 Mar 19;13:42. doi: 10.1186/1471-2105-13-42 (PMC3372431; doi:10.1186/1471-2105-13-42)
Supplement: Additional file 1 — Supplementary 1 Cloud BioLinux software documentation in the form of a mini, self-contained website. Users need to download and uncompress the .zip file, and open through a web browser the "index.html" file available on the main directory. (ZIP 1823 kb). [file 1471-2105-13-42-S1.ZIP › Cloud-BioLinux-Package-Documentation/docs/mummer.html]

Bio-Linux Software Documentation Pages

Back to search form

## mummer

|  |  |
| --- | --- |
| Name | mummer |
| Description | **mummer** is a part of the MUMmer package, for the rapid alignment of very large DNA and amino acid sequences.  **mummer** is a suffix tree algorithm designed to find maximal exact matches of some minimum length between two input sequences. MUMmer's namesake program originally stood for Maximal Unique Matcher, however in subsequent versions the meaning of unique has been skewed.  **References:**  Delcher AL, Kasif S, Fleischmann RD, Peterson J, White O, Salzberg SL: Alignment of whole genomes, Nucleic Acids Res. 1999 Jun 1;27(11):2369-76.[Entrez]    Delcher AL, Phillippy A, Carlton J, Salzberg SL: Fast algorithms for large-scale genome alignment and comparison, Nucleic Acids Res. 2002 Jun 1;30(11):2478-83.[Entrez]   Kurtz S, Phillippy A, Delcher AL, Smoot M, Shumway M, Antonescu C, Salzberg SL: Versatile and open software for comparing large genomes, Genome Biol. 2004;5(2):R12. Epub 2004 Jan 30.[Entrez] |
| Homepage | http://www.tigr.org/software/mummer/ |
| Remote Documentation | http://www.tigr.org/software/mummer/manual/ |
