# Supplementary material for: Cloud BioLinux: pre-configured and on-demand bioinformatics computing for the genomics community
Source: BMC Bioinformatics. 2012 Mar 19;13:42. doi: 10.1186/1471-2105-13-42 (PMC3372431; doi:10.1186/1471-2105-13-42)
Supplement: Additional file 1 — Supplementary 1 Cloud BioLinux software documentation in the form of a mini, self-contained website. Users need to download and uncompress the .zip file, and open through a web browser the "index.html" file available on the main directory. (ZIP 1823 kb). [file 1471-2105-13-42-S1.ZIP › Cloud-BioLinux-Package-Documentation/docs/build-icm.html]

Bio-Linux Software Documentation Pages

Back to search form

## build-icm

|  |  |
| --- | --- |
| Name | build-icm |
| Description | **build-icm** is a part of the Glimmer package, for finding genes in microbial DNA, especially the genomes of bacteria, archaea, and viruses.  This program reads (from stdin), the file generated by 'extract', which consists of a label followed by whitespace, then the open reading frame (orf) followed by a newline. The program then builds a Selective Markov Model using the following idea.  For each OFFSET of each orf, it determines the MAX\_DEPTH positions containing the greatest amount of mutual information after restricting certain bases to a particular position.  After determining where the maximum mutual information position is located, the node is printed to stdout (e.g. \_\_\_|\_\_\_|\*t\_ represents, using an INTERVAL of 9, that the next greatest mutual information position (denoted by '\*') is located in position 6, given base t is located in position 7 of the window.  Printed next are the counts and probabilities that position 8 is an a,c,t or g.  **References:**  Salzberg SL, Delcher AL, Kasif S, White O: Microbial gene identification using interpolated Markov models. Nucleic Acids Res. 1998 Jan 15;26(2):544-8. [Entrez]    Delcher AL, Harmon D, Kasif S, White O, Salzberg SL: Improved microbial gene identification with GLIMMER. Nucleic Acids Res. 1999 Dec 1;27(23):4636-41. [Entrez] |
| Homepage | http://www.tigr.org/software/glimmer/ |
| Remote Documentation | http://www.tigr.org/software/glimmer/glimmer.readme |
