# Supplementary material for: Cloud BioLinux: pre-configured and on-demand bioinformatics computing for the genomics community
Source: BMC Bioinformatics. 2012 Mar 19;13:42. doi: 10.1186/1471-2105-13-42 (PMC3372431; doi:10.1186/1471-2105-13-42)
Supplement: Additional file 1 — Supplementary 1 Cloud BioLinux software documentation in the form of a mini, self-contained website. Users need to download and uncompress the .zip file, and open through a web browser the "index.html" file available on the main directory. (ZIP 1823 kb). [file 1471-2105-13-42-S1.ZIP › Cloud-BioLinux-Package-Documentation/docs/priam.html]

Bio-Linux Software Documentation Pages

Back to search form

## priam

|  |  |
| --- | --- |
| Name | priam |
| Description | **PRIAM** is a program that searches for enzymes in a fully sequenced genome. The search is based on all sequences available in the ENZYME database.  **PRIAM** relies on sets of position-specific score matches (PSSMs) automatically tailored for each ENZYME entry.  **References:**  Clotilde Claudel-Renard, Claude Chavalet, Thomas Faraut and Daniel Kahn Enzyme-specific profiles for genome annotation: PRIAM Nucleic Acids Research, 2003, Vol. 31, No. 22 6633-6639 Entrez |
| Homepage |  |
| Remote Documentation |  |
