# Supplementary material for: Cloud BioLinux: pre-configured and on-demand bioinformatics computing for the genomics community
Source: BMC Bioinformatics. 2012 Mar 19;13:42. doi: 10.1186/1471-2105-13-42 (PMC3372431; doi:10.1186/1471-2105-13-42)
Supplement: Additional file 1 — Supplementary 1 Cloud BioLinux software documentation in the form of a mini, self-contained website. Users need to download and uncompress the .zip file, and open through a web browser the "index.html" file available on the main directory. (ZIP 1823 kb). [file 1471-2105-13-42-S1.ZIP › Cloud-BioLinux-Package-Documentation/docs/retree.html]

Bio-Linux Software Documentation Pages

Back to search form

## retree

|  |  |
| --- | --- |
| Name | retree |
| Description | **retree** is part of the PHYLIP package  Copyright 1993-2004 by The University of Washington. Written by Joseph Felsenstein. Permission is granted to copy this document provided that no fee is charged for it and that this copyright notice is not removed.  **retree** is a tree editor. It reads in a tree, or allows the user to construct one, and displays this tree on the screen. The user can then specify how the tree is to be rearranged, rerooted or written out to a file.  The input trees are in one file (with default file name intree), the output trees are written into another (outtree). The user can reroot, flip branches, change names of species, change or remove branch lengths, and move around to look at various parts of the tree if it is too large to fit on the screen. The trees can be multifurcating at any level, although the user is warned that many PHYLIP programs still cannot handle multifurcations above the root, or even at the root.  A major use for this program will be to change rootedness of trees so that a rooted tree derived from one program can be fed in as an unrooted tree to another (you are asked about this when you give the command to write out the tree onto the tree output file). It will also be useful for specifying the length of a branch in a tree where you want a program like DNAML, DNAMLK, FITCH, or CONTML to hold that branch length constant (see the L suboption of the User Tree option in those programs. It will also be useful for changing the order of species for purely cosmetic reasons for DRAWGRAM and DRAWTREE, including using the Midpoint method of rooting the tree. It can also be used to write out a tree file in the Nexus format used by Paup and MacClade or in our XML tree file format.  **References:**  Felsenstein, J. 1993. PHYLIP (Phylogeny Inference Package) version 3.5c. Distributed by the author. Department of Genetics, University of Washington, Seattle.    Felsenstein, J. 1989. PHYLIP -- Phylogeny Inference Package (Version 3.2). Cladistics 5: 164-166. |
| Homepage | http://evolution.genetics.washington.edu/phylip.html |
| Remote Documentation | http://evolution.genetics.washington.edu/phylip/doc/retree.html |
