# Supplementary material for: Cloud BioLinux: pre-configured and on-demand bioinformatics computing for the genomics community
Source: BMC Bioinformatics. 2012 Mar 19;13:42. doi: 10.1186/1471-2105-13-42 (PMC3372431; doi:10.1186/1471-2105-13-42)
Supplement: Additional file 1 — Supplementary 1 Cloud BioLinux software documentation in the form of a mini, self-contained website. Users need to download and uncompress the .zip file, and open through a web browser the "index.html" file available on the main directory. (ZIP 1823 kb). [file 1471-2105-13-42-S1.ZIP › Cloud-BioLinux-Package-Documentation/docs/maq.html]

Bio-Linux Software Documentation Pages

Back to search form

## maq

|  |  |
| --- | --- |
| Name | maq |
| Description | **Maq** builds mapping assemblies from short reads generated by new sequencing technologies - particularly data from Illumina-Solexa 1G Genetic Analyzer. Preliminary functions to handle ABI SOLiD data are also available. The Maq package is prepared by Debian-med and can be installed on Bio-Linux by typing: `sudo apt-get install maq` For further information about Maq, please refer to the remote documentation. |
| Homepage | http://maq.sourceforge.net |
| Remote Documentation | http://maq.sourceforge.net/maq-man.shtml |
