# Supplementary material for: Cloud BioLinux: pre-configured and on-demand bioinformatics computing for the genomics community
Source: BMC Bioinformatics. 2012 Mar 19;13:42. doi: 10.1186/1471-2105-13-42 (PMC3372431; doi:10.1186/1471-2105-13-42)
Supplement: Additional file 1 — Supplementary 1 Cloud BioLinux software documentation in the form of a mini, self-contained website. Users need to download and uncompress the .zip file, and open through a web browser the "index.html" file available on the main directory. (ZIP 1823 kb). [file 1471-2105-13-42-S1.ZIP › Cloud-BioLinux-Package-Documentation/docs/mcxdump.html]

Bio-Linux Software Documentation Pages

Back to search form

## mcxdump

|  |  |
| --- | --- |
| Name | mcxdump |
| Description |The **--dump-pairs** option yields a single matrix entry per line, identified by the respective column and row identifiers (either index or label) separated by the field separator.  
The **--dump-lines** and **--dump-rlines** result in the joining of all row entries on a single line, separated by the field separator. For both formats, the matrix value corresponding with a particular entry is by default output as well.   

| Homepage | http://micans.org/mcl |
| Remote Documentation | http://micans.org/mcl/man/distindex.html |

**mcxdump** reads a data file satisfying the mcl input format
