# Supplementary material for: Cloud BioLinux: pre-configured and on-demand bioinformatics computing for the genomics community
Source: BMC Bioinformatics. 2012 Mar 19;13:42. doi: 10.1186/1471-2105-13-42 (PMC3372431; doi:10.1186/1471-2105-13-42)
Supplement: Additional file 1 — Supplementary 1 Cloud BioLinux software documentation in the form of a mini, self-contained website. Users need to download and uncompress the .zip file, and open through a web browser the "index.html" file available on the main directory. (ZIP 1823 kb). [file 1471-2105-13-42-S1.ZIP › Cloud-BioLinux-Package-Documentation/docs/sim4.html]

Bio-Linux Software Documentation Pages

Back to search form

## sim4

|  |  |
| --- | --- |
| Name | sim4 |
| Description | sim4 is a similarity-based tool for aligning an expressed DNA sequence (EST, cDNA, mRNA) with a genomic sequence for a gene. sim4 also detects end matches when two input sequences overlap at one end (that is, the start of one sequence overlaps the end of the other).  **See also the sibsim4 program, more recently developed and based on sim4.** Sibsim4 is a substantial re-write of sim4 and may offer improved speed and functionality.  sim4 initially employs a blast-based technique to determine the basic matching blocks representing the "exon cores". In this first stage, it detects all possible exact matches of W-mers (i.e., DNA words of size W) between the two sequences and extends them to maximal scoring gap-free segments.  In the second stage, the exon cores are extended into the adjacent as-yet-unmatched fragments using greedy alignment algorithms, and heuristics are used to favour configurations that conform to the splice-site recognition signals (GT-AG, CT-AC). If necessary, the process is repeated with less stringent parameters on the unmatched fragments.  The sim4 package is prepared by Debian-Med and can be installed in Bio-Linux by typing `sudo apt-get install sim4` For further information please see the remote documentation links |
| Homepage | http://globin.cse.psu.edu/html/docs/sim4.html |
| Remote Documentation | http://globin.cse.psu.edu/html/docs/sim4.html |
