# Supplementary material for: Cloud BioLinux: pre-configured and on-demand bioinformatics computing for the genomics community
Source: BMC Bioinformatics. 2012 Mar 19;13:42. doi: 10.1186/1471-2105-13-42 (PMC3372431; doi:10.1186/1471-2105-13-42)
Supplement: Additional file 1 — Supplementary 1 Cloud BioLinux software documentation in the form of a mini, self-contained website. Users need to download and uncompress the .zip file, and open through a web browser the "index.html" file available on the main directory. (ZIP 1823 kb). [file 1471-2105-13-42-S1.ZIP › Cloud-BioLinux-Package-Documentation/docs/Qstats.html]

Bio-Linux Software Documentation Pages

Back to search form

## Qstats

|  |  |
| --- | --- |
| Name | Qstats |
| Description | **Qstats** is part of the QTL Cartographer suite of programs.  **Qstats** does some basic statistics on a dataset of quantitative traits. It plots a histogram and calculates the sample size, mean, variance standard deviation, skewness, kurtosis, and average deviation for a quantitative trait. The program also summarizes missing marker and trait data, as well as determining the marker types. Finally, **Qstats** will test whether markers are segregating at random. |
| Homepage | http://statgen.ncsu.edu/qtlcart/Qstats.php |
| Remote Documentation | http://statgen.ncsu.edu/qtlcart/Qstats.php |

Does some basic statistics on a dataset of quantitative traits.
