# Supplementary material for: Cloud BioLinux: pre-configured and on-demand bioinformatics computing for the genomics community
Source: BMC Bioinformatics. 2012 Mar 19;13:42. doi: 10.1186/1471-2105-13-42 (PMC3372431; doi:10.1186/1471-2105-13-42)
Supplement: Additional file 1 — Supplementary 1 Cloud BioLinux software documentation in the form of a mini, self-contained website. Users need to download and uncompress the .zip file, and open through a web browser the "index.html" file available on the main directory. (ZIP 1823 kb). [file 1471-2105-13-42-S1.ZIP › Cloud-BioLinux-Package-Documentation/docs/show-tiling.html]

Bio-Linux Software Documentation Pages

Back to search form

## show-tiling

|  |  |
| --- | --- |
| Name | show-tiling |
| Description | **show-tiling** is a part of the MUMmer package, for the rapid alignment of very large DNA and amino acid sequences.  **show-tiling** attempts to construct a tiling path out of the query contigs as mapped to the reference sequences. Given the delta alignment information of a few long reference sequences and many small query contigs, show-tiling will determine the best mapped location of each query contig. Note that each contig may only be tiled once, so repetitive regions may cause this program some difficulty. This program is useful for aiding in the scaffolding and closure of an unfinished set of contigs, if a suitable, high similarity reference genome is available. Or, if using PROmer, show-tiling will help in the identification of syntenic regions and their contig's mapping to the references.  **References:**  Delcher AL, Kasif S, Fleischmann RD, Peterson J, White O, Salzberg SL: Alignment of whole genomes, Nucleic Acids Res. 1999 Jun 1;27(11):2369-76.[Entrez]    Delcher AL, Phillippy A, Carlton J, Salzberg SL: Fast algorithms for large-scale genome alignment and comparison, Nucleic Acids Res. 2002 Jun 1;30(11):2478-83.[Entrez]    Kurtz S, Phillippy A, Delcher AL, Smoot M, Shumway M, Antonescu C, Salzberg SL: Versatile and open software for comparing large genomes, Genome Biol. 2004;5(2):R12. Epub 2004 Jan 30.[Entrez] |
| Homepage | http://www.tigr.org/software/mummer/ |
| Remote Documentation | http://www.tigr.org/software/mummer/manual/ |
