# Supplementary material for: Cloud BioLinux: pre-configured and on-demand bioinformatics computing for the genomics community
Source: BMC Bioinformatics. 2012 Mar 19;13:42. doi: 10.1186/1471-2105-13-42 (PMC3372431; doi:10.1186/1471-2105-13-42)
Supplement: Additional file 1 — Supplementary 1 Cloud BioLinux software documentation in the form of a mini, self-contained website. Users need to download and uncompress the .zip file, and open through a web browser the "index.html" file available on the main directory. (ZIP 1823 kb). [file 1471-2105-13-42-S1.ZIP › Cloud-BioLinux-Package-Documentation/docs/jprofilegrid.html]

Bio-Linux Software Documentation Pages

Back to search form

## jprofilegrid

|  |  |
| --- | --- |
| Name | jprofilegrid |
| Description | **JProfileGrid** is a java-based multiple sequence alignment tool that generates ProfileGrids for analysis and export. ProfileGrids are a new paradigm for concisely representing multiple sequence alignments for viewing and analysis.  For a protein sequence, a ProfileGrid matrix contains positional amino acid counts highlighted according to the frequency of the characters in the alignment.  JProfileGrid provides both command-line support and a graphical user interface. Please see the documentation files for a full description of the functionality of this software and the options available.  If JProfileGrid is not already on your Bio-Linux system, you can easily install it using a graphical package management tool such as the Ubuntu Software Centre - just search for jprofilegrid. Otherwise, you can type the following commands:  `sudo apt-get update sudo apt-get install bio-linux-jprofilegrid`  **References:**  A.I.Roca, A.E.Almada, and A.C.Abajian, ProfileGrids as a new visual representation of large multiple sequence alignments: a case study of the RecA protein family, (2008) BMC Bioinformatics 9: 554 |
| Homepage | http://www.profilegrid.org |
| Remote Documentation | http://www.profilegrid.org/examples.shtml      http://www.biomedcentral.com/1471-2105/9/554/abstract |
