# Supplementary material for: Cloud BioLinux: pre-configured and on-demand bioinformatics computing for the genomics community
Source: BMC Bioinformatics. 2012 Mar 19;13:42. doi: 10.1186/1471-2105-13-42 (PMC3372431; doi:10.1186/1471-2105-13-42)
Supplement: Additional file 1 — Supplementary 1 Cloud BioLinux software documentation in the form of a mini, self-contained website. Users need to download and uncompress the .zip file, and open through a web browser the "index.html" file available on the main directory. (ZIP 1823 kb). [file 1471-2105-13-42-S1.ZIP › Cloud-BioLinux-Package-Documentation/docs/drawgram.html]

Bio-Linux Software Documentation Pages

Back to search form

## drawgram

|  |  |
| --- | --- |
| Name | drawgram |
| Description | **drawgram** is a part of the PHYLIP package  Copyright 1990-2004 by The University of Washington. Written by Joseph Felsenstein. Permission is granted to copy this document provided that no fee is charged for it and that this copyright notice is not removed.  **drawgram** interactively plots a cladogram- or phenogram-like rooted tree diagram, with many options including orientation of tree and branches, style of tree, label sizes and angles, tree depth, margin sizes, stem lengths, and placement of nodes in the tree. Particularly if you can use your computer to preview the plot, you can very effectively adjust the details of the plotting to get just the kind of plot you want.  **References:**  Felsenstein, J. 1993. PHYLIP (Phylogeny Inference Package) version 3.5c. Distributed by the author. Department of Genetics, University of Washington, Seattle.    Felsenstein, J. 1989. PHYLIP -- Phylogeny Inference Package (Version 3.2). Cladistics 5: 164-166. |
| Homepage | http://evolution.genetics.washington.edu/phylip.html |
| Remote Documentation | http://evolution.genetics.washington.edu/phylip/doc/drawgram.html |
