# Supplementary material for: Cloud BioLinux: pre-configured and on-demand bioinformatics computing for the genomics community
Source: BMC Bioinformatics. 2012 Mar 19;13:42. doi: 10.1186/1471-2105-13-42 (PMC3372431; doi:10.1186/1471-2105-13-42)
Supplement: Additional file 1 — Supplementary 1 Cloud BioLinux software documentation in the form of a mini, self-contained website. Users need to download and uncompress the .zip file, and open through a web browser the "index.html" file available on the main directory. (ZIP 1823 kb). [file 1471-2105-13-42-S1.ZIP › Cloud-BioLinux-Package-Documentation/docs/dnacomp.html]

Bio-Linux Software Documentation Pages

Back to search form

## dnacomp

|  |  |
| --- | --- |
| Name | dnacomp |
| Description | **dnacomp** is part of the PHYLIP package  Copyright 1986-2004 by the University of Washington. Written by Joseph Felsenstein. Permission is granted to copy this document provided that no fee is charged for it and that this copyright notice is not removed.  This program implements the compatibility method for DNA sequence data. For a four-state character without a character-state tree, as in DNA sequences, the usual clique theorems cannot be applied. The approach taken in this program is to directly evaluate each tree topology by counting how many substitutions are needed in each site, comparing this to the minimum number that might be needed (one less than the number of bases observed at that site), and then evaluating the number of sites which achieve the minimum number. This is the evaluation of the tree (the number of compatible sites), and the topology is chosen so as to maximize that number.  **References:**  Felsenstein, J. 1993. PHYLIP (Phylogeny Inference Package) version 3.5c. Distributed by the author. Department of Genetics, University of Washington, Seattle.    Felsenstein, J. 1989. PHYLIP -- Phylogeny Inference Package (Version 3.2). Cladistics 5: 164-166. |
| Homepage | http://evolution.genetics.washington.edu/phylip.html |
| Remote Documentation | http://evolution.genetics.washington.edu/phylip/doc/dnacomp.html |
