# Supplementary material for: Cloud BioLinux: pre-configured and on-demand bioinformatics computing for the genomics community
Source: BMC Bioinformatics. 2012 Mar 19;13:42. doi: 10.1186/1471-2105-13-42 (PMC3372431; doi:10.1186/1471-2105-13-42)
Supplement: Additional file 1 — Supplementary 1 Cloud BioLinux software documentation in the form of a mini, self-contained website. Users need to download and uncompress the .zip file, and open through a web browser the "index.html" file available on the main directory. (ZIP 1823 kb). [file 1471-2105-13-42-S1.ZIP › Cloud-BioLinux-Package-Documentation/docs/dotter.html]

Bio-Linux Software Documentation Pages

Back to search form

## dotter

|  |  |
| --- | --- |
| Name | dotter |
| Description | **dotter** is a graphical tool for visualising the relationship between two sequences,which can be either DNA or protein. Dotter is a 'tool of best choice' for some problems, including locating and assesing repeats, and aligning proteins visually o­nto DNA. The sensitivity can be dynamically regulated, and is based o­n Karlin-Altschul statistics. Dotter requires an X11 display.  **dotter** is very useful for finding:   - overlaps between sequences. - overlap between contigs in a sequencing project. - visually identifying repeat sequences within a sequence. - overlaps with known sequences (eg. vectors, transposons, other genes etc).  *Why is dotter a good dotplot program?*  - A threshold above which a dot is plotted can be set interactively. This allows the user to vary the signal/noise ratio easily. In many other dotplot programs, you have to re-run the program each time you re-define the threshold value. - Dotter shows a sliding alignment of your sequences. If you move the cursor within the dotplot output, the sequences in the alignment box re-align accordingly (easier to see than to explain!). - Dotter can be run, and the output saved to be viewed later. - You can compare a number of sequences with each other simultaneously using dotter. This is particularly useful if you are looking at a series of sequences and looking for overlap between them, or repeats within them.  The command line usage for dotter is in the following format:  `dotter [options] query_seq subject_seq [X options]`  *Format of the query and subject sequences must be in Fasta or raw format.*  Options on the command can be:   - -b Batch mode, write dotplot to -l Load dotplot from -m Memory usage limit in Mb (default 0.5) - -z Set zoom (compression) factor - -p Set pixel factor manually (ratio pixelvalue/score) - -W Set sliding window size. (K => Karlin/Altschul estimate) - -M Read in score matrix from (Blast format; Default: Blosum62) - -f Read feature segments from -i Do NOT use installed private colormap, but share with other apps - -r Reverse and complement horizontal\_sequence (DNA vs Protein) - -D Don't display mirror image in self comparisons - -w For DNA: horizontal\_sequence top strand o­nly (Watson) - -c For DNA: horizontal\_sequence bottom strand o­nly (Crick) - -q Horizontal\_sequence offset - -s Vertical\_sequence offset  The most important X options are:   - -acefont < font> Main font. - -font < font> Menu font.  (Any standard X option can also be used, such as -bg green -fg red.)  Example command line usage:    run dotter to compare sequence1.tfa and sequence2.tfa  **dotter sequence1.tfa sequence2.tfa**    run dotter and save the results to results.file  **dotter -bresults.file sequence1.tfa sequence2.tfa**    View results.file  **dotter -lresults.file sequence1.tfa sequence2.tfa**  More information o­n using dotter: Dotter's Home and UVic, Canada  Related resources from the Sonhammer group see: http://sonnhammer.sbc.su.se/databases.html  **References:**  A dot-matrix program with dynamic threshold control suited for genomic DNA and protein sequence analysis Erik L.L. Sonnhammer and Richard Durbin Gene 167:GC1-10 (1995) [Entrez] |
| Homepage | http://www.cgr.ki.se/cgb/groups/sonnhammer/Dotter.html |
| Remote Documentation | http://www.cgr.ki.se/cgr/groups/sonnhammer/Dotter.html |
