# Supplementary material for: Cloud BioLinux: pre-configured and on-demand bioinformatics computing for the genomics community
Source: BMC Bioinformatics. 2012 Mar 19;13:42. doi: 10.1186/1471-2105-13-42 (PMC3372431; doi:10.1186/1471-2105-13-42)
Supplement: Additional file 1 — Supplementary 1 Cloud BioLinux software documentation in the form of a mini, self-contained website. Users need to download and uncompress the .zip file, and open through a web browser the "index.html" file available on the main directory. (ZIP 1823 kb). [file 1471-2105-13-42-S1.ZIP › Cloud-BioLinux-Package-Documentation/docs/showpep.html]

Bio-Linux Software Documentation Pages

Back to search form

## showpep

|  |  |
| --- | --- |
| Name | showpep |
| Description | **showpep** displays one or more protein sequences, with features, in a style suitable for publication. The output is sent to screen by default but can be written to file. You may pick a format from a list, alternatively, use the many options to control what is output and in what format. Optionally, the sequence feature table can be displayed. There are various other options for controlling how the sequence is displayed and numbered and the output can be formatted for HTML.    **showpep -h** for basic programme options    **showpep -h -v** for further programme options     **tfm showpep** for full program informations |
| Homepage | http://emboss.sourceforge.net |
| Remote Documentation | http://emboss.sourceforge.net/apps/release/6.3/emboss/apps/showpep.html |

EMBOSS documentation for showpep
