# Supplementary material for: Cloud BioLinux: pre-configured and on-demand bioinformatics computing for the genomics community
Source: BMC Bioinformatics. 2012 Mar 19;13:42. doi: 10.1186/1471-2105-13-42 (PMC3372431; doi:10.1186/1471-2105-13-42)
Supplement: Additional file 1 — Supplementary 1 Cloud BioLinux software documentation in the form of a mini, self-contained website. Users need to download and uncompress the .zip file, and open through a web browser the "index.html" file available on the main directory. (ZIP 1823 kb). [file 1471-2105-13-42-S1.ZIP › Cloud-BioLinux-Package-Documentation/docs/mapview.html]

Bio-Linux Software Documentation Pages

Back to search form

## mapview

|  |  |
| --- | --- |
| Name | mapview |
| Description | **mapview** is a part of the MUMmer package, for the rapid alignment of very large DNA and amino acid sequences.  **MapView** is a utility script for displaying sequence alignments as provided by NUCmer or PROmer. It takes the output from show-coords or mgaps and converts it to a FIG, PDF or PS image file. By default, it produces FIG files which can be viewed with the common system utility xfig or converted to PDF or PS with the fig2dev utility (neither programs are included with MUMmer).  **mapview** is useful for mapping multiple query contigs (e.g. from a draft sequencing project) against an annotated reference sequence. Exons and other features can also be plotted with the NUCmer or PROmer alignments, aiding in exon refinement and analysis. Individual MUMmer hits are plotted according to their percent identity, making regions of high or low similarity easily distinguishable.  **References:**  Delcher AL, Kasif S, Fleischmann RD, Peterson J, White O, Salzberg SL: Alignment of whole genomes, Nucleic Acids Res. 1999 Jun 1;27(11):2369-76.[Entrez]    Delcher AL, Phillippy A, Carlton J, Salzberg SL: Fast algorithms for large-scale genome alignment and comparison, Nucleic Acids Res. 2002 Jun 1;30(11):2478-83.[Entrez]    Kurtz S, Phillippy A, Delcher AL, Smoot M, Shumway M, Antonescu C, Salzberg SL: Versatile and open software for comparing large genomes, Genome Biol. 2004;5(2):R12. Epub 2004 Jan 30.[Entrez] |
| Homepage | http://www.tigr.org/software/mummer/ |
| Remote Documentation | http://www.tigr.org/software/mummer/manual/ |

HTML manual for the Mummer program
