# Supplementary material for: Cloud BioLinux: pre-configured and on-demand bioinformatics computing for the genomics community
Source: BMC Bioinformatics. 2012 Mar 19;13:42. doi: 10.1186/1471-2105-13-42 (PMC3372431; doi:10.1186/1471-2105-13-42)
Supplement: Additional file 1 — Supplementary 1 Cloud BioLinux software documentation in the form of a mini, self-contained website. Users need to download and uncompress the .zip file, and open through a web browser the "index.html" file available on the main directory. (ZIP 1823 kb). [file 1471-2105-13-42-S1.ZIP › Cloud-BioLinux-Package-Documentation/docs/prot4est.html]

Bio-Linux Software Documentation Pages

Back to search form

## prot4est

|  |  |
| --- | --- |
| Name | prot4est |
| Description | **prot4eEST** takes expressed sequence tags (ESTs) and translates them into putative peptide sequences. It incorporates the ESTScan program and optionally the decoder program if this available on the machine.  **References:**  Wasmuth JD, Blaxter ML. BMC Bioinformatics. 2004 Nov 30;5(1):187 |
| Homepage | http://zeldia.cap.ed.ac.uk/bioinformatics/ |
| Remote Documentation | http://zeldia.cap.ed.ac.uk/PartiGene/downloads/README.p4e |
