# Supplementary material for: Cloud BioLinux: pre-configured and on-demand bioinformatics computing for the genomics community
Source: BMC Bioinformatics. 2012 Mar 19;13:42. doi: 10.1186/1471-2105-13-42 (PMC3372431; doi:10.1186/1471-2105-13-42)
Supplement: Additional file 1 — Supplementary 1 Cloud BioLinux software documentation in the form of a mini, self-contained website. Users need to download and uncompress the .zip file, and open through a web browser the "index.html" file available on the main directory. (ZIP 1823 kb). [file 1471-2105-13-42-S1.ZIP › Cloud-BioLinux-Package-Documentation/docs/cytoscape.html]

Bio-Linux Software Documentation Pages

Back to search form

## cytoscape

|  |  |
| --- | --- |
| Name | cytoscape |
| Description | **Cytoscape** is a popular, well-regarded, open source software platform for visualizing complex-networks and integrating these with any type of attribute data. We highly recommend you refer to the  for full information on this piece of software.  Many plugins are available for various kinds of problem domains, including bioinformatics, social network analysis, and semantic web. You can install these when running Cytoscape on your system by going to the Plugins menu. If you install a plugin, it will be put into your home directory, under the folder `~/.cytoscape/2.7/plugins`. This means that each user can have their own set of plugins installed. |
| Homepage | http://www.cytoscape.org |
| Remote Documentation | http://www.cytoscape.org/documentation\_users.html |
