# Supplementary material for: Cloud BioLinux: pre-configured and on-demand bioinformatics computing for the genomics community
Source: BMC Bioinformatics. 2012 Mar 19;13:42. doi: 10.1186/1471-2105-13-42 (PMC3372431; doi:10.1186/1471-2105-13-42)
Supplement: Additional file 1 — Supplementary 1 Cloud BioLinux software documentation in the form of a mini, self-contained website. Users need to download and uncompress the .zip file, and open through a web browser the "index.html" file available on the main directory. (ZIP 1823 kb). [file 1471-2105-13-42-S1.ZIP › Cloud-BioLinux-Package-Documentation/docs/blastclust.html]

Bio-Linux Software Documentation Pages

Back to search form

## blastclust

|  |  |
| --- | --- |
| Name | blastclust |
| Description | **blasclust** automatically and systematically clusters protein or DNA sequences based on pairwise matches found using the BLAST algorithm in case of proteins, or the megablast algorithm in the case of nucleotide sequence.  **blastclust** is one of the programs distributed along with the old blastall program by the NCBI. The NCBI recommends that people start using the programs of the **blast+** package instead. Having said that, most documentation currently available still refers to blastall and the programs distributed with it.  The programs distributed with blastall include:  - bl2seq - blast2 - blastall - blastcl3 - blastclust - blastpgp - copymat - fastacmd - formatdb - formatrpsdb - impala - makemat - megablast - rpsblast - seedtop - taxblast  The man pages for these provide further details. |
| Homepage | http://www.ncbi.nlm.nih.gov/BLAST/ |
| Remote Documentation | http://www.ncbi.nlm.nih.gov/Education/BLASTinfo/info      http://www.ncbi.nlm.nih.gov/staff/tao/URLAPI/blastclust.html |
