# Supplementary material for: Cloud BioLinux: pre-configured and on-demand bioinformatics computing for the genomics community
Source: BMC Bioinformatics. 2012 Mar 19;13:42. doi: 10.1186/1471-2105-13-42 (PMC3372431; doi:10.1186/1471-2105-13-42)
Supplement: Additional file 1 — Supplementary 1 Cloud BioLinux software documentation in the form of a mini, self-contained website. Users need to download and uncompress the .zip file, and open through a web browser the "index.html" file available on the main directory. (ZIP 1823 kb). [file 1471-2105-13-42-S1.ZIP › Cloud-BioLinux-Package-Documentation/docs/partigene.html]

Bio-Linux Software Documentation Pages

Back to search form

## partigene

|  |  |
| --- | --- |
| Name | partigene |
| Description | **PartiGene** is an integrated sequence analysis suite which uses freely available public domain software to :    - process raw trace chromatograms into sequence objects suitable for submission to dbEST; - place these sequences within a genomic context; - perform customisable annotation of the data; - present the data as HTML tables and an SQL database resource. |
| Homepage | http://nema.cap.ed.ac.uk/PartiGene/ |
| Remote Documentation | http://envgen.nox.ac.uk/envgen/software/egtdc\_dev/PartiGene2.1/PartiGeneUGv2.1.pdf |
