# Supplementary material for: Cloud BioLinux: pre-configured and on-demand bioinformatics computing for the genomics community
Source: BMC Bioinformatics. 2012 Mar 19;13:42. doi: 10.1186/1471-2105-13-42 (PMC3372431; doi:10.1186/1471-2105-13-42)
Supplement: Additional file 1 — Supplementary 1 Cloud BioLinux software documentation in the form of a mini, self-contained website. Users need to download and uncompress the .zip file, and open through a web browser the "index.html" file available on the main directory. (ZIP 1823 kb). [file 1471-2105-13-42-S1.ZIP › Cloud-BioLinux-Package-Documentation/docs/rasmol.html]

Bio-Linux Software Documentation Pages

Back to search form

## rasmol

|  |  |
| --- | --- |
| Name | rasmol |
| Description | **RasMol** is a molecular graphics program intended for the visualisation of proteins, nucleic acids and small molecules. The program is aimed at display, teaching and generation of publication quality images.  **RasMol** reads in a molecule coordinate file and interactively displays the molecule on �the screen in a variety of colour schemes and molecule representations. Currently available representations include depth-cued wireframes, Dreiding sticks, spacefilling (CPK) spheres, ball and stick, solid and strand biomolecular ribbons, atom labels and dot surfaces.  **References:**  Shanthi V, Selvarani P, Kumar ChK, Mohire CS, Sekar K. SSEP: Secondary structural elements of proteins Nucleic Acids Res. 2003 Jul 1;31(13):3404-5. |
| Homepage | http://www.openrasmol.org/ |
| Remote Documentation | http://www.openrasmol.org/doc/rasmol.html |
