# Supplementary material for: Cloud BioLinux: pre-configured and on-demand bioinformatics computing for the genomics community
Source: BMC Bioinformatics. 2012 Mar 19;13:42. doi: 10.1186/1471-2105-13-42 (PMC3372431; doi:10.1186/1471-2105-13-42)
Supplement: Additional file 1 — Supplementary 1 Cloud BioLinux software documentation in the form of a mini, self-contained website. Users need to download and uncompress the .zip file, and open through a web browser the "index.html" file available on the main directory. (ZIP 1823 kb). [file 1471-2105-13-42-S1.ZIP › Cloud-BioLinux-Package-Documentation/docs/clmmate.html]

Bio-Linux Software Documentation Pages

Back to search form

## clmmate

|  |  |
| --- | --- |
| Name | clmmate |
| Description | **clmmate** computes for each cluster C1 in clfile1 all clusters C2 in clfile2 that have non-empty intersection, and prints the number of shared nodes, the numbers of nodes in C1 absent in C2, the number of nodes in C2 absent in C1, and the total number of nodes in C2.  The total number of nodes in C1 is listed once for each sublisting, where the primary listing is over clusters in clfile1. |
| Homepage | http://micans.org/mcl |
| Remote Documentation | http://micans.org/mcl/man/distindex.html |
