# Supplementary material for: Cloud BioLinux: pre-configured and on-demand bioinformatics computing for the genomics community
Source: BMC Bioinformatics. 2012 Mar 19;13:42. doi: 10.1186/1471-2105-13-42 (PMC3372431; doi:10.1186/1471-2105-13-42)
Supplement: Additional file 1 — Supplementary 1 Cloud BioLinux software documentation in the form of a mini, self-contained website. Users need to download and uncompress the .zip file, and open through a web browser the "index.html" file available on the main directory. (ZIP 1823 kb). [file 1471-2105-13-42-S1.ZIP › Cloud-BioLinux-Package-Documentation/docs/pregap4.html]

Bio-Linux Software Documentation Pages

Back to search form

## pregap4

|  |  |
| --- | --- |
| Name | pregap4 |
| Description | **Pregap4** is part of the Staden package of sequence assembly, editing and analysis tools. **Pregap4** provides a **graphical user interface** to set up the processing required to prepare trace data for assembly or analysis.  The possible processes which can be set up via pregap4 include trace format conversion, quality analysis, vector clipping, contaminant screening, mutation detection and searching for repeats.  See also infomation about gap4, spin and trev. |
| Homepage | http://sourceforge.net/projects/staden/ |
| Remote Documentation | http://staden.sourceforge.net/ |
