# Supplementary material for: Cloud BioLinux: pre-configured and on-demand bioinformatics computing for the genomics community
Source: BMC Bioinformatics. 2012 Mar 19;13:42. doi: 10.1186/1471-2105-13-42 (PMC3372431; doi:10.1186/1471-2105-13-42)
Supplement: Additional file 1 — Supplementary 1 Cloud BioLinux software documentation in the form of a mini, self-contained website. Users need to download and uncompress the .zip file, and open through a web browser the "index.html" file available on the main directory. (ZIP 1823 kb). [file 1471-2105-13-42-S1.ZIP › Cloud-BioLinux-Package-Documentation/docs/restml.html]

Bio-Linux Software Documentation Pages

Back to search form

## restml

|  |  |
| --- | --- |
| Name | restml |
| Description | **restml** is part of the PHYLIP package  Copyright 1986-2004 by The University of Washington. Written by Joseph Felsenstein. Permission is granted to copy this document provided that no fee is charged for it and that this copyright notice is not removed.  This program implements a maximum likelihood method for restriction sites data (not restriction fragment data). This program is one of the slowest programs in this package, and can be very tedious to run. It is possible to have the program search for the maximum likelihood tree. It will be more practical for some users (those that do not have fast machines) to use the U (User Tree) option, which takes less run time, optimizing branch lengths and computing likelihoods for particular tree topologies suggested by the user.  The model used here is essentially identical to that used by Smouse and Li (1987) who give explicit expressions for computing the likelihood for three-species trees. It does not place prior probabilities on trees as they do. The present program extends their approach to multiple species by a technique which, while it does not give explicit expressions for likelihoods, does enable their computation and the iterative improvement of branch lengths. It also allows for multiple restriction enzymes. The algorithm has been described in a paper (Felsenstein, 1992). Another relevant paper is that of DeBry and Slade (1985).  **References:**  Felsenstein, J. 1993. PHYLIP (Phylogeny Inference Package) version 3.5c. Distributed by the author. Department of Genetics, University of Washington, Seattle.    Felsenstein, J. 1989. PHYLIP -- Phylogeny Inference Package (Version 3.2). Cladistics 5: 164-166. |
| Homepage | http://evolution.genetics.washington.edu/phylip.html |
| Remote Documentation | http://evolution.genetics.washington.edu/phylip/doc/restml.html |
