# Supplementary material for: Cloud BioLinux: pre-configured and on-demand bioinformatics computing for the genomics community
Source: BMC Bioinformatics. 2012 Mar 19;13:42. doi: 10.1186/1471-2105-13-42 (PMC3372431; doi:10.1186/1471-2105-13-42)
Supplement: Additional file 1 — Supplementary 1 Cloud BioLinux software documentation in the form of a mini, self-contained website. Users need to download and uncompress the .zip file, and open through a web browser the "index.html" file available on the main directory. (ZIP 1823 kb). [file 1471-2105-13-42-S1.ZIP › Cloud-BioLinux-Package-Documentation/docs/fasts.html]

Bio-Linux Software Documentation Pages

Back to search form

## fasts

|  |  |
| --- | --- |
| Name | fasts |
| Description | **fasts** is part of the *Fasta3* package.  **fasts** compares short peptide fragments, as would be obtained from mass-spec. analysis of a protein, against a protein database. See also tfasts that allows comparison to a DNA database and fastf, which compares mixed peptides to a protein sequence database.   In the Bio-Linux package, the threaded versions of the **fasta** programs are the default.   The programs available in the *Fasta3* package are:   - fasta - scan a protein or DNA sequence library for similar sequences. - fastx - compare a DNA sequence to a protein sequence database, comparing the translated DNA sequence in forward and reverse frames. - tfastx - compare a protein sequence to a DNA sequence database, calculating similarities with frameshifts to the forward and reverse orientations. - fasty - compare a DNA sequence to a protein sequence database, comparing the translated DNA sequence in forward and reverse frames.- tfasty - compare a protein sequence to a DNA sequence database, calculating similarities with frameshifts to the forward and reverse orientations.   - fasts - compare unordered peptides to a protein sequence database- tfasts - compare unordered peptides to a translated DNA sequence database- fastm - compare ordered peptides (or short DNA sequences) to a protein (DNA) sequence database- fastm - compare ordered peptides (or short DNA sequences) to a translated DNA sequence database- fastf - compare mixed peptides to a protein sequence database- tfastf - compare mixed peptides to a translated DNA sequence database             - ssearch - compare a protein or DNA sequence to a sequence database using the Smith-Waterman algorithm.             - ggsearch - compare a protein or DNA sequence to a sequence database using a global alignment (Needleman-Wunsch)- lalign - produce multiple non-overlapping alignments for protein and DNA sequences using the Huang and Miller SIM algorithm for the Waterman-Eggert algorithm. This version of **lalign** replaces that from the Fasta2 package.               - **prss** - (discontinued, replaced in the fasta35 release by new versions of ssearch and fastx) estimate statistical significance of an alignment by comparing the score to the distribution of similarity scores generated by shuffling the second sequence. **prss35** uses Smith-Waterman. **prfx35** uses the **fastx** algorithm.   **References:**  Aaron J. Mackey, Timothy A. J. Haystead, and William R. Pearson. Algorithms for Rapid Protein Identification with Multiple Short Peptide Sequences. Mol Cell Proteomics 2002 1: 139-147 Full text    Pearson, W.R. Flexible sequence similarity searching with the FASTA3 program package. Methods Mol Biol. 2000;132:185-219 [Entrez]    Pearson, W.R. Empirical statistical estimates for sequence similarity searches. J Mol Biol. 1998 Feb 13;276(1):71-84 [Entrez]    Pearson WR, Wood T, Zhang Z, Miller W. Comparison of DNA sequences with protein sequences. Genomics. 1997 Nov 15;46(1):24-36. [Entrez] |
| Homepage | http://www.people.virginia.edu/~wrp/pearson.html |
| Remote Documentation | http://www.people.virginia.edu/~wrp/papers/ismb2000.pdf |
