# Supplementary material for: Cloud BioLinux: pre-configured and on-demand bioinformatics computing for the genomics community
Source: BMC Bioinformatics. 2012 Mar 19;13:42. doi: 10.1186/1471-2105-13-42 (PMC3372431; doi:10.1186/1471-2105-13-42)
Supplement: Additional file 1 — Supplementary 1 Cloud BioLinux software documentation in the form of a mini, self-contained website. Users need to download and uncompress the .zip file, and open through a web browser the "index.html" file available on the main directory. (ZIP 1823 kb). [file 1471-2105-13-42-S1.ZIP › Cloud-BioLinux-Package-Documentation/docs/JZmapqtl.html]

Bio-Linux Software Documentation Pages

Back to search form

## JZmapqtl

|  |  |
| --- | --- |
| Name | JZmapqtl |
| Description | **JZmapqtl** is part of the QTL Cartographer suite of programs.  **JZmapqtl** uses (composite) interval mapping to map quantitative trait loci to a map of molecular markers and can analyze multiple traits simultaneously. It requires a molecular map that could be a random one produced by **Rmap**, or a real one in the same format as the output of **Rmap**.  In addition, the program requires the results of the stepwise linear regression analysis of **SRmapqtl** for composite interval mapping. |
| Homepage | http://statgen.ncsu.edu/qtlcart/JZmapqtl.php |
| Remote Documentation | http://statgen.ncsu.edu/qtlcart/JZmapqtl.php |

Uses (composite) interval mapping to map quantitative trait loci to a map of molecular markers and can analyze multiple traits simultaneously.
