# Supplementary material for: Cloud BioLinux: pre-configured and on-demand bioinformatics computing for the genomics community
Source: BMC Bioinformatics. 2012 Mar 19;13:42. doi: 10.1186/1471-2105-13-42 (PMC3372431; doi:10.1186/1471-2105-13-42)
Supplement: Additional file 1 — Supplementary 1 Cloud BioLinux software documentation in the form of a mini, self-contained website. Users need to download and uncompress the .zip file, and open through a web browser the "index.html" file available on the main directory. (ZIP 1823 kb). [file 1471-2105-13-42-S1.ZIP › Cloud-BioLinux-Package-Documentation/docs/fastdnaml.html]

Bio-Linux Software Documentation Pages

Back to search form

## fastdnaml

|  |  |
| --- | --- |
| Name | fastdnaml |
| Description | **fastDNAml** is a program for estimating maximum likelihood phylogenetic trees from nucleotide sequences. Much of this program is based o�n version 3.3 of Joseph Felsenstein's DNAML program (this is to give him credit, not to have you contact him for information o�n fastDNAml). This program implements the maximum likelihood method for DNA sequences. The present version is faster than earlier versions of DNAML. Details of the algorithm are published in the paper by Felsenstein and Churchill (1996). The model of base substitution allows the expected frequencies of the four bases to be unequal, allows the expected frequencies of transitions and transversions to be unequal, and has several ways of allowing different rates of evolution at different sites.  **References:**  Olsen, G. J., Matsuda, H., Hagstrom, R., and Overbeek, R. 1994. fastDNAml: A tool for construction of phylogenetic trees of DNA sequences using maximum likelihood. Comput. Appl. Biosci. 10: 41-48 [Entrez]    Felsenstein, J. 1981. Evolutionary trees from DNA sequences: A maximum likelihood approach. J. Mol. Evol. **17**: 368-376. |
| Homepage | http://geta.life.uiuc.edu/~gary/programs/fastDNAml.html |
| Remote Documentation | http://geta.life.uiuc.edu/~gary/programs/fastDNAml/fastDNAml\_1.2.2/docs/ |
