# Supplementary material for: Cloud BioLinux: pre-configured and on-demand bioinformatics computing for the genomics community
Source: BMC Bioinformatics. 2012 Mar 19;13:42. doi: 10.1186/1471-2105-13-42 (PMC3372431; doi:10.1186/1471-2105-13-42)
Supplement: Additional file 1 — Supplementary 1 Cloud BioLinux software documentation in the form of a mini, self-contained website. Users need to download and uncompress the .zip file, and open through a web browser the "index.html" file available on the main directory. (ZIP 1823 kb). [file 1471-2105-13-42-S1.ZIP › Cloud-BioLinux-Package-Documentation/docs/QTL_Cartographer.html]

Bio-Linux Software Documentation Pages

Back to search form

## QTL\_Cartographer

|  |  |
| --- | --- |
| Name | QTL\_Cartographer |
| Description | **QTL Cartographer** is a suite of programs for mapping quantitative trait loci (QTLs) onto a genetic linkage map.  This package consists of several programs that perform various tasks, including simulating, reformatting or analyzing data and visualizing the results of the analyses.  All input and output files are plain text.  The programs in the package include:   - Emap- Rmap- Rqtl- Rcross- Qstats- LRmapqtl- SRmapqtl- Zmapqtl- JZmapqtl- MImapqtl- MultiRegress- Prune- Preplot- Eqtl |
| Homepage | http://statgen.ncsu.edu/qtlcart |
| Remote Documentation | http://statgen.ncsu.edu/qtlcart/manual/ |
