# Supplementary figures and images for: Cloud BioLinux: pre-configured and on-demand bioinformatics computing for the genomics community
Source: BMC Bioinformatics. 2012 Mar 19;13:42. doi: 10.1186/1471-2105-13-42 (PMC3372431; doi:10.1186/1471-2105-13-42)

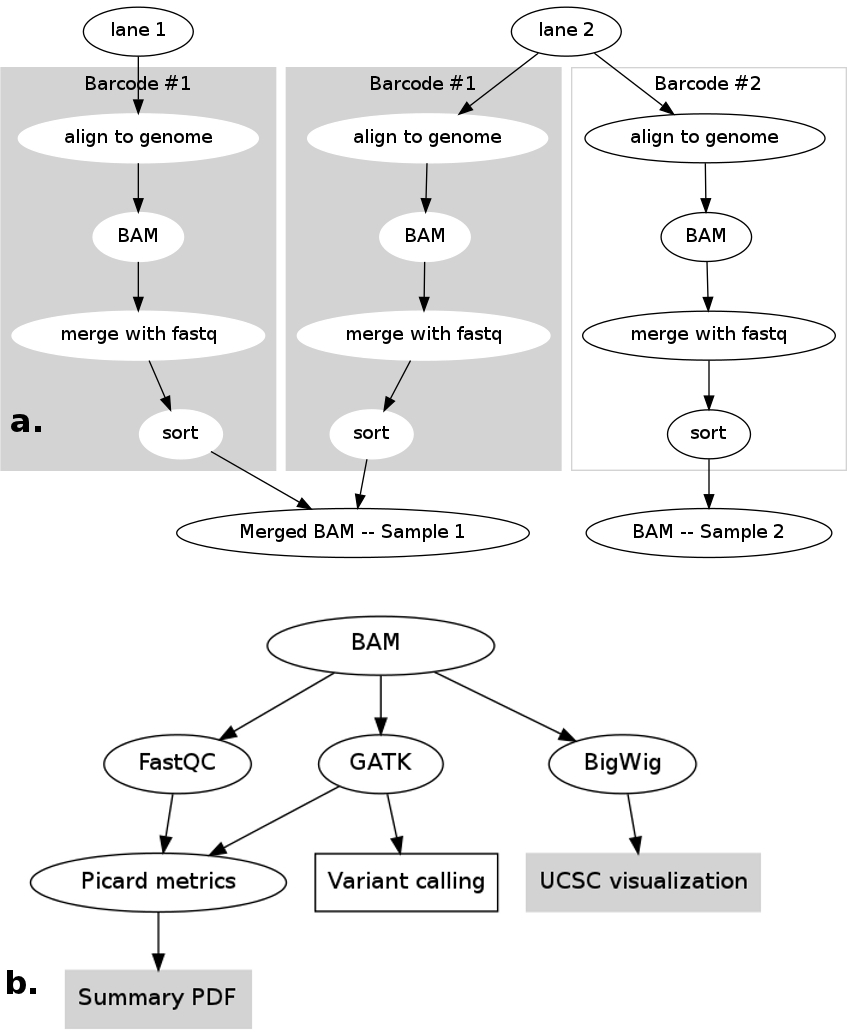

Supplement: Additional file 2 — Supplementary 2. Overall, the pipeline takes as input sequencing reads, converts them to standard Fastq format, aligning to a reference genome, doing SNP calling, and producing a summary PDF of results. Furthermore, it leverages the CloudMan server that is included in Cloud BioLinux VM for provisioning a Sun Grid Engine (SGE) cluster composed of multiple copies of the VM on the cloud, and the RabbitMQ distributed messaging queue for data coordination and exchange between the cluster nodes respectively (a.) sequence files for each lane are split based on the sample barcodes and the parts are aligned using Bowtie to the genome in parallel, by being submitted as separate SGE computational tasks across the cluster nodes. The pipeline then sorts and merges BAM alignment files from multiple lanes if sequences have the same barcode, producing a single representative BAM file for each barcoded sub-sample. At this step, RabbitMQ enables message exchange between the cluster nodes in order to identify BAM sequence alignment sets with the same barcode on different nodes, and the pipeline scripts transfer and merge those sets on a single node (b.) the second phase parallelizes the processing of each alignment file with read quality assessment, variant calling and visualization, using an identical approach based on SGE parallel computation across cluster nodes, with the software tools performing each of these steps being respectively Fastqc and Picard, GATK and BigWig. (JPEG 160 kb). [file 1471-2105-13-42-S2.JPEG]
